# Oncolytic vaccinia virus as a vector for therapeutic sodium iodide symporter gene therapy in prostate cancer

**DOI:** 10.1038/gt.2016.5

**Published:** 2016-02-18

**Authors:** D C Mansfield, J N Kyula, N Rosenfelder, J Chao-Chu, G Kramer-Marek, A A Khan, V Roulstone, M McLaughlin, A A Melcher, R G Vile, H S Pandha, V Khoo, K J Harrington

**Affiliations:** 1 Divisions of Cancer Biology and Radiotherapy and Imaging, The Institute of Cancer Research, Chester Beatty Labs, London, UK; 2 Leeds Institute of Cancer and Pathology, University of Leeds, St James's University Hospital, Leeds, UK; 3 Molecular Medicine Program, Mayo Clinic, Rochester, MN, USA; 4 Postgraduate Medical School, The University of Surrey, Guildford, UK; 5 The Royal Marsden Hospital, London, UK; 6 University of Melbourne and Monash University, Victoria, Australia

## Abstract

Oncolytic strains of vaccinia virus are currently in clinical development with clear evidence of safety and promising signs of efficacy. Addition of therapeutic genes to the viral genome may increase the therapeutic efficacy of vaccinia. We evaluated the therapeutic potential of vaccinia virus expressing the sodium iodide symporter (NIS) in prostate cancer models, combining oncolysis, external beam radiotherapy and NIS-mediated radioiodide therapy. The NIS-expressing vaccinia virus (VV-NIS), GLV-1h153, was tested in *in vitro* analyzes of viral cell killing, combination with radiotherapy, NIS expression, cellular radioiodide uptake and apoptotic cell death in PC3, DU145, LNCaP and WPMY-1 human prostate cell lines. *In vivo* experiments were carried out in PC3 xenografts in CD1 nude mice to assess NIS expression and tumor radioiodide uptake. In addition, the therapeutic benefit of radioiodide treatment in combination with viral oncolysis and external beam radiotherapy was measured. *In vitro* viral cell killing of prostate cancers was dose- and time-dependent and was through apoptotic mechanisms. Importantly, combined virus therapy and iodizing radiation did not adversely affect oncolysis. NIS gene expression in infected cells was functional and mediated uptake of radioiodide both *in vitro* and *in vivo*. Therapy experiments with both xenograft and immunocompetent Transgenic Adenocarcinoma of the Mouse Prostate (TRAMP) mouse models showed that the addition of radioiodide to VV-NIS-infected tumors was more effective than each single-agent therapy, restricting tumor growth and increasing survival. In conclusion, VV-NIS is effective in prostate cancer models. This treatment modality would be an attractive complement to existing clinical radiotherapy practice.

## Introduction

The sodium iodide symporter (NIS) membrane protein is responsible for the uptake of iodide by the thyroid tissue. In cases of papillary and follicular thyroid cancer, radioactive Iodine-131 (^131^I) is used to ablate residual normal (and malignant) thyroid tissue after thyroidectomy, with high rates of efficacy.^1^ Targeted delivery of the NIS gene as a mode of cancer gene therapy represents an opportunity to bring the proven therapeutic potential of I^131^ to bear against other cancer types.

Oncolytic virotherapy exploits the natural or engineered affinity of certain viral strains selectively to infect and replicate in cancer cells and, in doing so, to kill them. In clinical testing, they have been shown to have excellent safety profiles and promising signs of efficacy.^2, 3^ The adenovirus H101 has been approved in China for use in head/neck cancer.^4^ A positive phase III trial of talimogene laherperepvec (T-Vec, herpes simplex virus type 1) has been reported^5^ and this agent received approval by the European Medicines Agency in October 2015 for patients with malignant melanoma.^6^

Vaccinia virus belongs to the *Poxviridae* family and possesses a large linear double-stranded DNA genome consisting of ~250 genes, with capacity for insertion of therapeutic transgenes, such as the NIS gene.^7^ Vaccinia has been administered widely as the smallpox vaccine and, as such, it has an excellent safety profile.^8^ It has also been examined extensively in attenuated forms as an oncolytic agent with comparable safety.^9^ The complex life cycle of Vaccinia includes dual mechanisms of infection by separate forms of infectious particles. Intracellular mature virions are the main product of viral lysis and extracellular enveloped virions are actively shed by infected cells.^10^ Compared with other agents, Vaccinia offers a number of potential advantages including rapid replication in and lysis of infected cells, the ability to achieve high levels of viral gene expression, the capacity to spread cell-to-cell and the fact that its activity is unhindered by hypoxia^11^ and therapeutic irradiation.^12^ Genetic modification of Vaccinia to express the NIS gene represents a further refinement of its therapeutic potential by giving it the capacity to drive cellular ^131^I uptake for direct killing of infected cells and indirect killing of neighboring cells within the 0.8 mm range of the emitted β particles.^13^ Previous studies have explored the potential of NIS, delivered by a range of oncolytic viruses including measles, HSV and VSV as a therapeutic reporter gene and as a therapeutic agent.^14, 15, 16, 17, 18^ Oncolytic vaccinia virus has been studied *in vitro* in a range of tumor types, enabling positron emission tomography and single photon emission computed tomography observation of viral kinetics using a variety of radioiosotopes including ^131^I, ^124^I and ^99m^Tc.^19, 20, 21^ This has been shown to be a viable imaging method in a phase I/II trial of measles virus strains encoding NIS in ovarian cancer patients^18^ and would be a useful safety-monitoring tool to confirm that viral biodistribution in other human trials is as expected. Furthermore, oncolytic vaccinia enabled NIS therapy has shown additional benefit of ^131^I administration in pancreatic and breast cancer models.^21, 22^

Prostate cancer is the commonest form of male cancer and the second highest cause of cancer death in the United States, with ~240 000 new cases and 28 000 deaths annually.^23^ Currently, prostate cancer treatment typically involves radical prostatectomy or radiotherapy with good survival outcomes for the 90% of patients whose disease is diagnosed at the local/regional stage.^1^ However, the side-effects of such treatments can be significant and include incontinence, bowel complications and erectile dysfunction, with associated long-term detriment to quality of life. The prognosis for those patients who develop castration resistant disease is poor.^24^ Despite recent advances in medical therapies,^25, 26, 27, 28^ men with prostate cancer will ultimately develop treatment-refractory, incurable disease. Therefore, there is a need for novel therapies with improved side-effect profiles in locoregional disease and improved efficacy in metastatic disease. Prostate cancer has been targeted for NIS gene therapy in numerous pre-clinical studies.^29^ Using adenovirus as a vector, NIS gene expression in prostate tissue has, in a Phase 1 trial, proven the 99mTc-imaging approach to be both safe and feasible.^30^ Further study is ongoing.^31^ To date, no human trials have studied the potential of oncolytic viral therapy to additionally enable NIS ^131^I therapy.

In this study, we examine the therapeutic potential of the NIS-expressing Vaccinia virus (VV-NIS), GLV-1h153 (VV-NIS), as an oncolytic agent and as a vector for targeting NIS gene therapy to prostate cancer cells *in vitro* and *in vivo*. We demonstrate efficient time- and dose-dependent marker gene expression and oncolysis of a panel of human prostate cell lines and confirm that these virally mediated effects are not affected by combination with radiotherapy. Combined GLV-1h153 and radiation therapy is shown to enhance apoptosis of prostate cancer cells. NIS is shown to be both expressed and functional, enabling the prostate cells to concentrate radioiodide. *In vivo* NIS gene expression and iodide uptake is demonstrated in xenograft tumors of PC3 cells and therapeutic experiments show the combination of virus and ^131^I to be significantly more effective against these tumors than either therapy alone. In the immunocompetent Transgenic Adenocarcinoma of the Mouse Prostate (TRAMP) model of prostate cancer this radio-virotherapeutic approach also proved to be effective.

## Results

### GLV-1h153 cytotoxicity in prostate cancer cell lines

**Figure 1:**
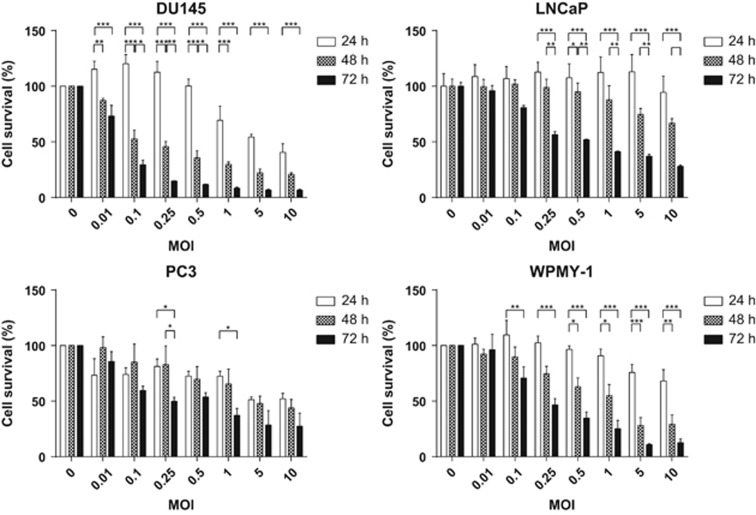
Prostate cancer cells were infected with GLV-1h153 and the reduction of proliferative capability was measured by MTT assay at 24, 48 and 72 h post infection. s.e.m.s are shown. Significance is the result of two-way ANOVA with Bonferroni multiple comparisons test, *P<0.05, **P<0.001, ***P<0.0001.

A panel of four prostate cancer cell lines, DU145, PC3, LNCaP and WPMY-1, was tested for their susceptibility to the GLV-1h153 virus. The cells were infected with virus at multiplicities of infection (MOI) ranging between 0.0001 and 10 for periods ranging from 24 to 72 h. Reduction in cell proliferation relative to uninfected controls was then assessed by 3-(4,5-dimethylthiazol-2-yl)-2,5-diphenyltetrazolium bromide (MTT) assay. The virus was effective against all four cell lines, killing in a dose- and time-dependent manner (two-way analysis of variance (ANOVA) *P*<0.0001 for both virus and time factors as sources of variation for each cell line individually) (Figure 1). DU145, LNCaP and WPMY-1 showed a positive interaction between time and dose (two-way ANOVA *P*<0.0001), producing increased cell death over time. PC3 were somewhat resistant to this effect, especially at MOI 0.001–0.1.

### NIS mRNA expression in GLV-1h153-infected cells

The cell lines DU145, PC3 and WPMY-1 were infected with GLV-1h153 MOIs ranging between 0.001 and 1 for 24 h before levels of NIS mRNA were quantified by quantitative reverse transcriptase PCR. Levels of mRNA detected correlated positively with increasing MOI of the virus (Supplementary Figure 1). There was no NIS mRNA detectable in the uninfected cells.

### *In vitro*^131^I uptake in GLV-1h153-infected cells

**Figure 2:**
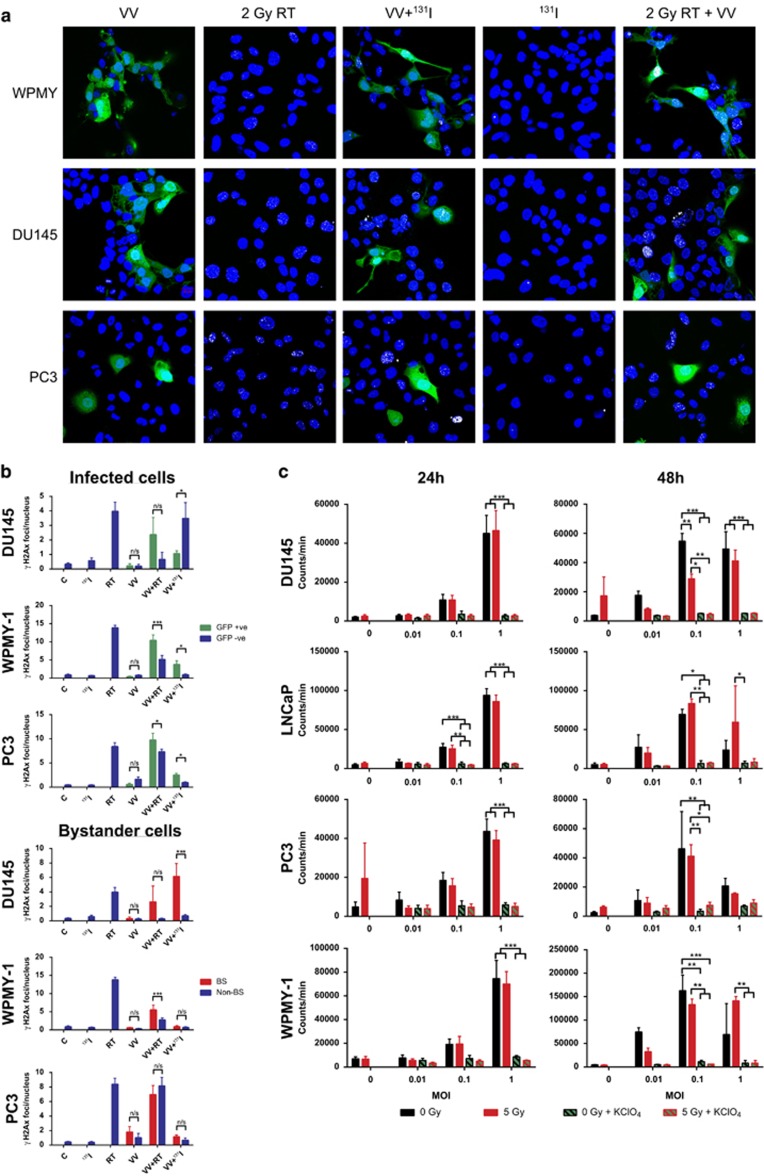
Functional assays were used to measure viral NIS expression. (**a**) Representative confocal images of the effect of GLV-1h153 infection (VV), 2 Gy external beam radiotherapy (2 Gy RT), Combined GLV-1h153 infection and radioiodide treatment (VV+^131^I), radioiodide treatment alone (^131^I) and combined GLV-1h153 and 2 Gy external beam radiotherapy (2 Gy RT+VV) on DNA double-strand breaks measured by γH2Ax foci in WPMY (Top) and DU145 (Middle) and PC3 (Bottom) cells. Blue: DAPI, Green: Viral GFP, White: H2Ax foci. White arrows mark the non-infected ‘bystander' cells that have received DNA damage. (**b**) Quantification of confocal images examining the distribution of H2Ax foci among subsets of cells. Untreated control (**c**), radioiodide treated (^131^I), 2 Gy external beam radiotherapy (RT), GLV-1h153 infection (VV) and combinations thereof. s.e.s are shown. Significance is the result of two-Way ANOVA with Sidak's multiple comparisons test, *P<0.05, **P<0.001, ***P<0.0001. (**c**) Radioiodide uptake following GLV-1h153 infection at a range of multiplicities of infection (MOI), with and without the influence of external beam radiation (5 Gy) and potassium perchlorate (KClO_4_). s.e.m.s are shown. Significance is the result of two-Way ANOVA with Bonferroni multiple comparisons test, *P<0.05, **P<0.001, ***P<0.0001.

Confocal microscopy was used to look directly at WPMY-1, PC3 and DU145 cells during infection to determine the levels of H2Ax foci (DNA double-strand break marker) induced by either viral infection or ^131^I uptake (Figure 2a). Cells irradiated with 2 Gy exhibited H2Ax foci, whereas those incubated with ^131^I showed very few. However, when infected cells were incubated with ^131^I many more H2Ax foci were observed. These images were then quantified to compare levels of DNA damage in sub-groups of cells (Figure 2b). First, infected cells, determined by green fluorescent protein (GFP) expression, were compared with non-infected cells. Infected WPMY-1 and PC3 cells were more susceptible to DNA damage by external beam irradiation. DU145 showed a similar profile although this was not statistically significant. In infected DU145 cells incubated with ^131^I, non-infected cells had a greater number of H2Ax foci, though this was not seen in WPMY-1 and PC3, where infected cells had a greater proportion of foci. With infected cells accumulating iodide and appearing to cause an increase in DNA damage, it was hypothesized that uninfected cells closer to infected ones should receive a greater dose of radiation than those further away, according to the inverse square law of radiation. We hypothesized that this would account for uninfected DU145 cells receiving a greater proportion of the ^131^I-induced DNA damage than the infected cells. To evaluate this, the confocal images were again quantified, with a perimeter of 20 μm around infected cells in which any other cell nuclei would be classed as a ‘bystander', cells wholly outside the perimeter were ‘non-bystanders' and infected cells were excluded. The bystander DU145 cells exhibited sixfold more H2Ax foci than non-bystanders when exposed to ^131^I. This effect was also seen to a lesser extent with external beam radiation, suggesting that infection somehow radiosensitises the non-infected DU145 cells, though this was not statistically significant. WPMY cells did show a significant radio-senitization effect on bystander cells, whereas PC3 did not. No significant bystander effect was observed in the WPMY or PC3 bystander cells exposed to ^131^I, although in these cell lines there were few foci in non-infected cells. As another, more general, measure of bystander effect, the images were quantified to compare the total number of H2Ax foci in the field of view to the total number of infected cells (Supplementary Figure 2). Comparison by this method revealed a positive correlation between number of infected cells and H2Ax foci in DU145 cells, and no such correlation in WPMY-1 and PC3, thus supporting the above data.

To verify the functional expression of the NIS transgene, *in vitro* iodide uptake assays were performed. Cells were irradiated with 5 Gy (or not) and then infected at MOIs of 0.01, 0.1 and 1 for 24 or 48 h. At this time, they were treated with ^131^I and radioiodide uptake was measured by gamma emissions from the cells. At 24 h, all four cell lines showed NIS expression and iodide uptake at MOI 1, with a highly significant difference (two-way ANOVA *P*<0.001) compared with cells treated with the competitive inhibitor of iodide uptake (potassium perchlorate) (Figure 2c). LNCaP also had significant uptake with MOI 0.1 at 24 h (two-way ANOVA *P* <0.01). At 48 h, all four cell lines showed an increase in iodide uptake at MOI 0.1 compared with that seen at 24 h. Cells irradiated with 5 Gy showed little difference in the levels of iodide uptake except in DU145 at 48 h where the level of uptake at an MOI of 0.1 was significantly lower.

### GLV-1h153 cytotoxicity in combination with iodizing radiation

**Figure 3:**
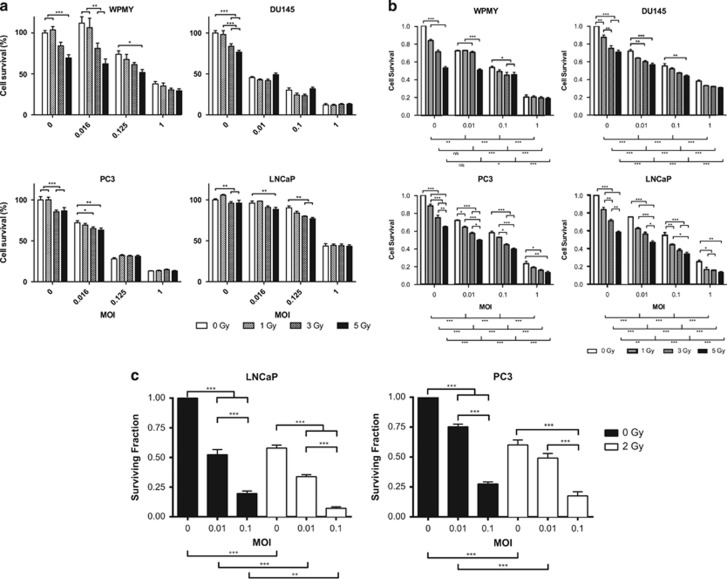
Prostate cancer cells treated with GLV-1h153 4 h after external beam irradiation. Reduction of proliferative capability measured by: (**a**) MTT assay at 72 h; and (**b**) SRB cytotoxicity assay at 48 h. s.e.m.s are shown. Significance is the result of two-way ANOVA with Bonferroni multiple comparisons test, *P<0.05, **P<0.001, ***P<0.0001. (**c**) Clonogenic capacity of cells treated with GLV-1h153 and external beam radiation. Significance is the result of one-way ANOVA with Tukey's multiple comparisons test, *P<0.05, **P<0.001, ***P<0.0001.

To confirm that iodizing radiation does not affect the ability of the virus to replicate, express transgenes and cause cell death in prostate cells, external beam irradiation was used as a substitute for ^131^I to allow controlled and equal exposure of all cells to iodizing radiation. The range from 1 to 5 Gy was selected to represent radiation doses that would not immediately kill the cells and should allow viral infection to be productive and potentially show synergy as previously published in other models.^12, 32^ The panel of cell lines was infected with MOIs ranging between 0.01 and 1, either 4 h pre- or post-irradiation with 1, 3 or 5 Gy. Cells were incubated for periods of 24, 48 and 72 h and cell proliferation was assessed by 3-[4,5-dimethylthiazol-2-yl]-2,5 diphenyl tetrazolium bromide (MTT) assay. Cells irradiated prior to infection all showed a significant dose-dependent effect of virus at all time points (two-way ANOVA *P*<0.0001). Irradiation had a significant dose-dependent effect in LNCaP and WPMY-1 at 24 and 48 h; LNCaP, WPMY-1 and PC3 at 48 h (Supplementary Figure 3); and all four cell lines at 72 h (two-way ANOVA *P*<0.0001) (Figure 3a). Irradiation never significantly reduced the viral effect. The alternative scheduling, irradiation 4 h post infection, yielded broadly similar results and indicated no benefit of one schedule over the other (Supplementary Figure 4).

The sulforhodamine B assay was also used to confirm the results observed with the MTT assay. Radiation was delivered 4 h pre-infection and cell number was measured at 48 h. These data also showed a significant radiation dose effect (two-way ANOVA *P*<0.0001) and confirmed that the viral effect is unaffected by irradiation (Figure 3b).

Clonogenic assays were performed with the two cell lines that were capable of colony formation (LNCaP and PC3) in order to determine the longer-term effects of the virus-radiation combination on cell survival. These data show that the clonogenic potential of cells was reduced by irradiation and by viral infection in dose-dependent fashion (for each agent). The combination of the two treatments reduced the clonogenic capacity of the cells to a greater extent than either treatment alone (Figure 3c).

### Expression of viral transgenes in combination with iodizing radiation

**Figure 4:**
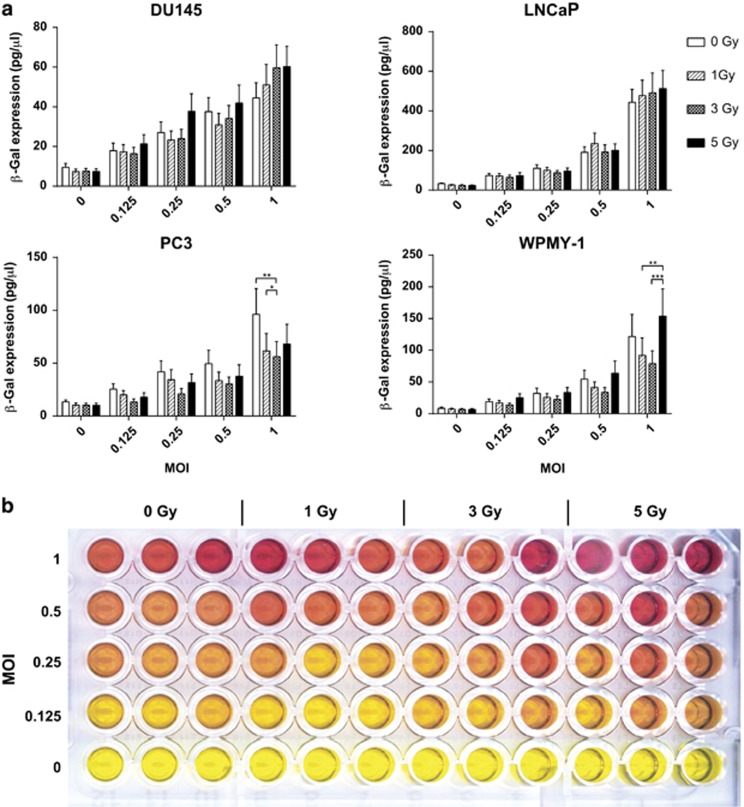
Viral gene expression in GLV-1h153-infected cells treated with external beam radiotherapy. (**a**) Viral β-galactosidase measured by CPRG assay at a range of MOI and radiation doses 24 h after treatment. s.em.s are shown. Significance is the result of two-way ANOVA with Bonferroni multiple comparisons test, *P<0.05, **P<0.001, ***P<0.0001. (**b**) Representative example of the colorimetric CPRG assay. Red shows viral β-galactosidase activity.

Chlorophenolred-β-D-galactopyranoside assay was used to confirm that viral transgene expression was not adversely affected by irradiation. The cell line panel was irradiated with between 1 and 5 Gy 4 h prior to infection with MOIs ranging between 0.016 and 1. β-galactosidase gene expression was measured at 24, 48 and 72 h. At 24 h (Figures 4a and b), all four cell lines showed statistically significant viral dose-dependent expression of β–galactosidase (two-way ANOVA *P*<0.0001) and no consistent significant irradiation effect (positive or negative) on gene expression. Gene expression levels at MOI 1 were greatest with LNCaP (994 pg μl^−1^), followed by WPMY-1 (275 pg μl^−1^), PC3 (150 pg μl^−1^) and DU145 (103 pg μl^−1^). At 48 and 72 h (Supplementary Figure 5) the gene expression continued to increase. At 72 h gene expression at the highest MOI had dropped relative to the 48 h time point, correlating with the cell death shown in Figure 1. The inverse treatment schedule of GLV-1h153 infection 4 h prior to irradiation showed a similar gene expression profile (Supplementary Figure 6).

### Induction of apoptotic cell death by GLV-1h153 and iodizing radiation

**Figure 5:**
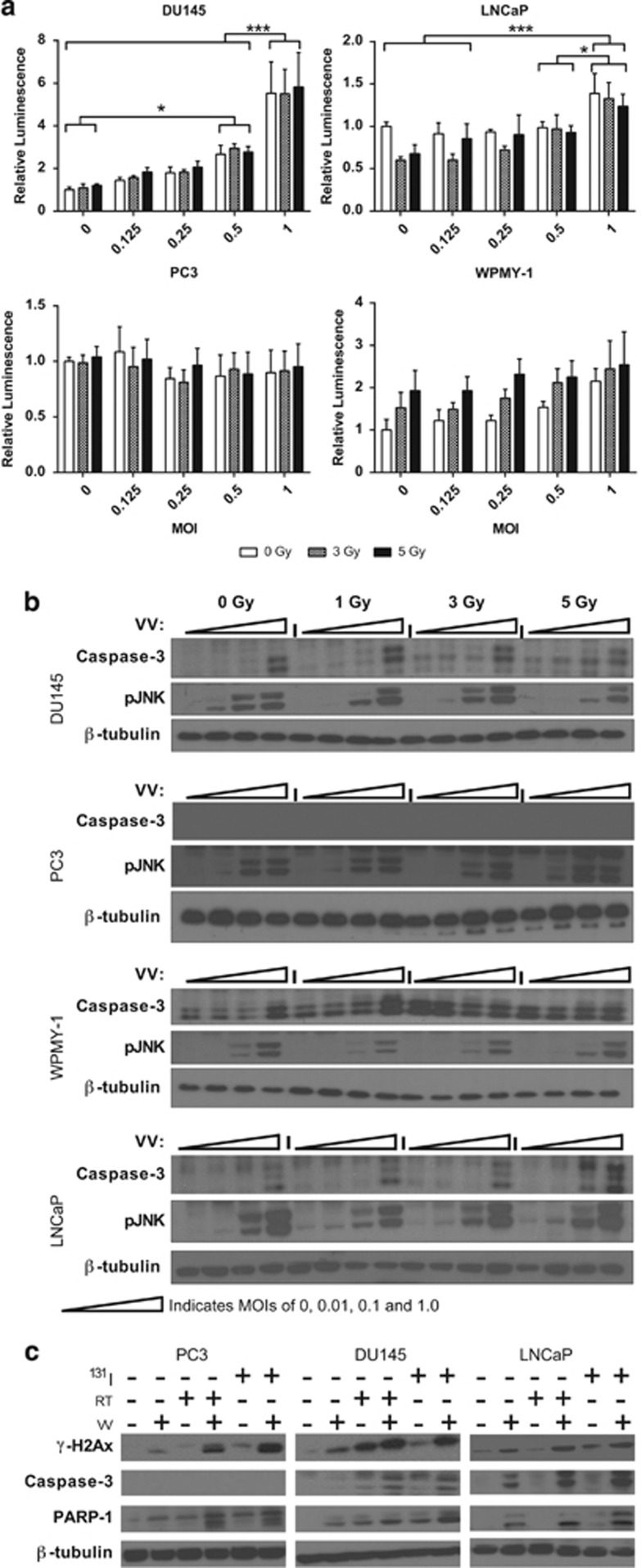
Mechanisms of cell death in cells treated with GLV-1h153 and radiation. (**a**) Caspase-3/7 activity 48 h post treatment. s.e.m.s are shown. Significance is the result of two-way ANOVA with Bonferroni multiple comparisons test, *P<0.05, ***P<0.0001. (**b**) Activation of apoptosis by cleavage of caspase-3 and phosphorylation of JNK in response to external beam radiation and GLV-1h153 infection at MOIs of 0, 0.01, 0.1 and 1. (**c**) Induction of apoptosis in response to GLV-1h153 infection (MOI 0.1), external beam radiotherapy (2 Gy), ^131^I (5 μCi) and combinations thereof, measured by γH2Ax expression, caspase-3 cleavage and PARP-1 cleavage.

The panel of cell lines was analyzed for the apoptotic response to virus at MOIs between 0.01 and 1 and irradiation between 1 and 5 Gy. The response varied widely between cell lines (Figure 5a). By Caspase Glo3/7 assay, DU145 showed strong (up to 5.5-fold) Caspase-3/7 induction in response to virus (two-way ANOVA *P*<0.0001), but no significant radiation effect was observed. LNCaP also had a small but significant response (up to 1.4-fold) to virus only (two-way ANOVA *P*<0.0001). PC3 had no Caspase-3/7 response to either virus or irradiation; and WPMY had a significant response (up to twofold) to irradiation only (two-way ANOVA *P*=0.0137).

Western blots for the active cleaved form of Caspase-3 correlated closely with the Caspase-Glo data, showing no functional Caspase-3 in PC3 cells and replicating the patterns of sensitivity observed in the remaining cell lines. It has previously been published that synergy between radiation and vaccinia virus in BRAF mutant melanoma is dependent on signaling via JNK and TNF-α.^32^ Phospho-JNK western blots showed that JNK becomes highly active in response to virus in all cell lines, though no clear radiation effect was observed (Figure 5b).

Combination of viral infection and irradiation consistently activated markers of apoptosis to high levels, with the combination of viral infection and ^131^I showing higher levels of PARP-1 cleavage than the combination with external beam radiation in DU145 and LNCaP (Figure 5c and Supplementary Figure 7). The effects were approximately equal in PC3. Despite PC3 seemingly lacking functional Caspase-3, cleavage of PARP-1 and expression of γH2Ax both indicate that apoptosis is occurring in this cell line.

### *In vivo* viral gene expression and ^131^I uptake in GLV-1h153-infected tumors

**Figure 6:**
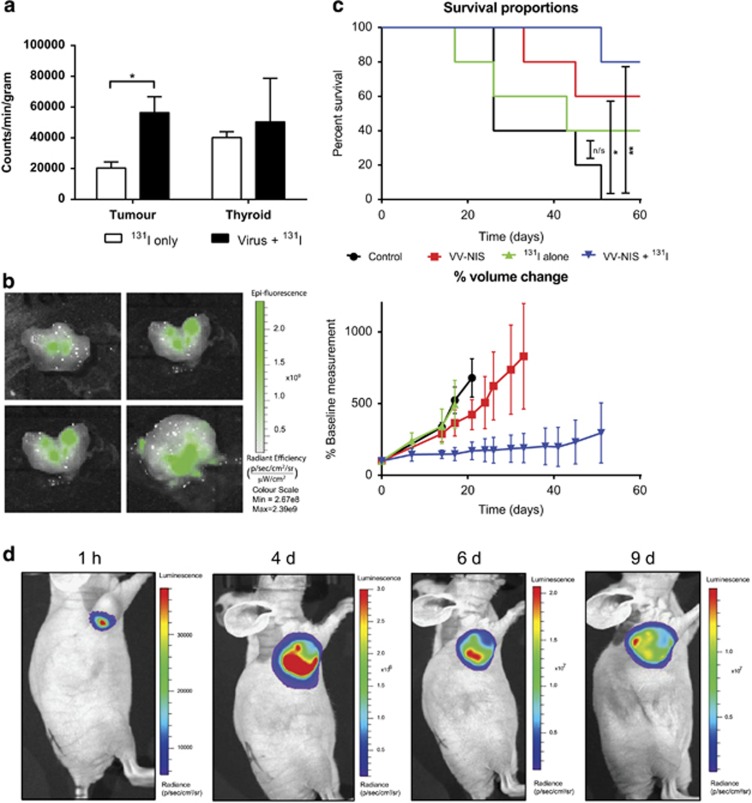
*In vivo* GLV-1h153 gene expression and therapy. (**a**) Gamma emissions from excised PC3 xenografts treated with intratumoural injection of 1 × 10^6^ PFU GLV-1h153 and 1 mCi of 131I 48 h later, alongside controls that received ^131^I only. (**b**) Viral GFP expression in intratumourally treated xenografts. (**c**) Long-term therapeutic effect of treatment with GLV-1h153 and ^131^I on PC3 xenografts. Kaplan–Meier plot significance is the result of log-rank (Mantel Cox) test. *P<0.05, **P<0.001. Tumor volume plot shows s.e.m.s. (**d**) Biodistribution of viral gene expression detected by viral encoded Renilla luciferase bioluminescence following intratumoural administration of GLV-1h153 in PC3 xenograft.

The PC3 cell line was selected for *in vivo* experiments, as it had high levels of viral gene expression persisting for 72 h post infection *in vitro*. Xenografts in CD1 nu/nu mice were allowed to grow to 5–10 mm in diameter before intratumoural injection of virus (1 × 10^6^ plaque forming units (PFU)) or control injection. After 48 h, ^131^I was administered intraperitoneally as described. A further 48 h later, tumor iodide uptake and GFP expression were measured (Figures 6a and b). Tumors injected with virus had gamma emissions 2.7-fold higher than control tumors (*t*-test *P*<0.05). There was no significant difference in emissions from thyroid tissue between the two treatment groups. In tumors injected with virus, GFP expression was observed at low levels. Bioluminescence generated by viral Renilla luciferase indicated that viral replication and gene expression is limited to the tumor tissue only (Figure 6d). Uptake of ^131^I in non-NIS-expressing tissue was minimal, with tumor tissue uptake being twice that in the blood (Supplementary Figure 8).

### GLV-1h153 and ^131^I combined treatment in PC3 xenografts

PC3 xenografts in CD1 nu/nu mice were allowed to grow to 5–10 mm in diameter. Mice were divided into four groups, two of which received intratumoural injection of virus. After 48 h, one treated group and one non-treated group received ^131^I. Tumors treated with ^131^I only continued to grow at the same rate as the controls (Log-Rank *P*=0.41). Virus alone slowed growth marginally and significantly improved survival to 60% at study termination, 60 days post treatment (Log-Rank *P*<0.05). The virus and ^131^I treatment combination stabilised tumor growth and improved survival at study termination to 80% versus 0% in the control group (Log-Rank *P*=0.004), with all surviving mice having stable or completely regressed tumors (Figure 6c).

### GLV-1h153 biodistribution from PC3 xenografts

PC3 xenografts in CD1 nu/nu mice were allowed to grow to ~10 mm in diameter and then received intratumoural injection of virus (1 × 10^6^ PFU). At 1 h and at 4, 6 and 9 days post virus administration 100 μg coelenterazine, the substrate for the viral encoded Renilla luciferase, was administered to the animals to allow bioluminescent detection of areas of viral gene expression (Figure 6d and Supplementary Figure 9). These images show that viral replication was limited to the tumor tissue only and did not disseminate to other tissues. Low levels of gene expression were detected in 75% of the animals at the 1 h time point, and the level of gene expression increased markedly over the following time points.

### GLV-1h153 in TRAMP-C cell lines

Following the positive results from the PC3 model in immunodeficient CD1 nu/nu mice, we considered that a fully immunocompetent model would more closely reflect the challenges faced by the virus in a clinical setting. The TRAMP transgenic model of murine prostate cancer was selected.

**Figure 7:**
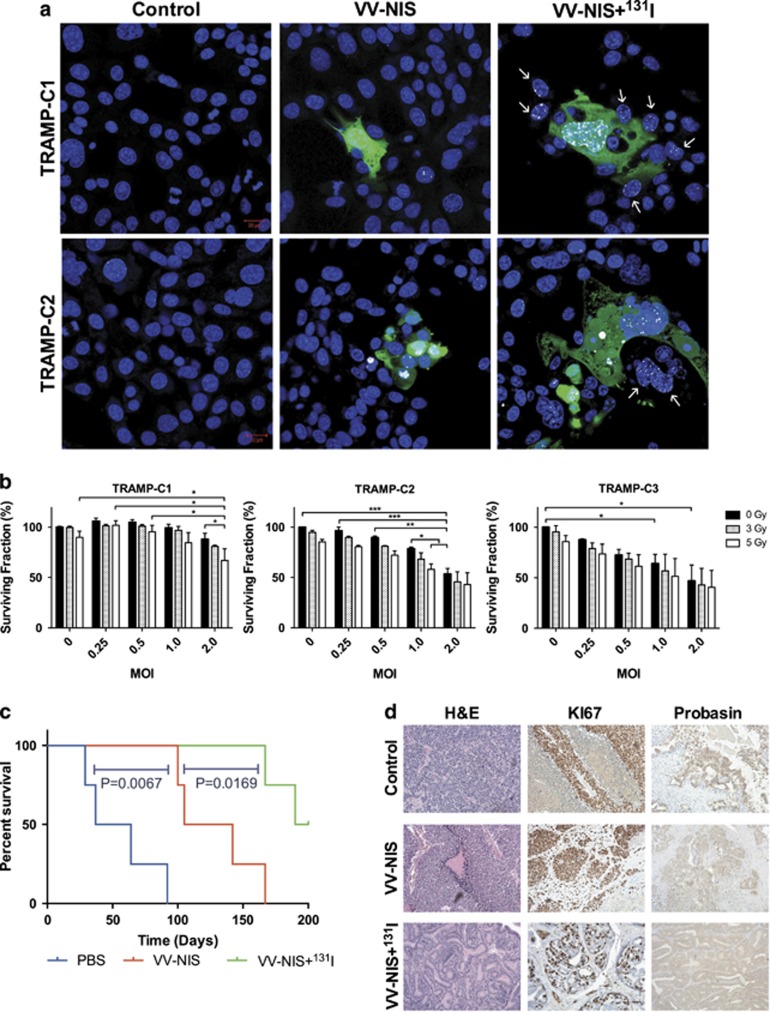
Efficacy of GLV-1h153 in TRAMP models. (**a**) Confocal images of H2Ax foci resulting from DNA double-strand breaks in TRAMP cells treated with GLV-1h153 and ^131^I. Blue: DAPI, Green: Viral GFP, White: γH2Ax foci. White arrows mark the non-infected ‘bystander' cells that have received DNA damage. (**b**) TRAMP cells treated with GLV-1h153 and external beam radiation. Reduction of proliferative capability was measured by MTT assay at 48 h post infection. s.e.m.s are shown. Significance is the result of two-way ANOVA with Bonferroni multiple comparisons test, *P<0.05, **P<0.001, ***P<0.0001. (**c**) Long-term survival of TRAMP mice bearing spontaneous prostate tumors and treated intravenously with 5 × 10^7^ PFU GLV-1h153 (VV-NIS) and 1 mCi ^131^I (VV-NIS+^131^I) (*n*=4). Kaplan–Meier plot significance is the result of log-rank (Mantel Cox) test. (**d**) Representative examples of the histology of the above TRAMP mouse prostates, showing H&E staining and IHC for KI67 and probasin at the time of death.

To test the species specificity of GLV-1h153, three cell lines derived from TRAMP tumors (TRAMP-C1, -C2, -C3) were challenged with the virus. The TRAMP-C cell lines were susceptible to infection and viral GFP expression was observed 24 h post infection (Figure 7a, Supplementary Figure 10). DNA damage due to radioiodide uptake was also seen in both infected cells and bystander cells.

In cell proliferation assays (Figure 7b), the TRAMP-C1 line showed some resistance to viral infection and a slower death as compared to the data for the human cell lines at 48 h post infection. Tramp-C2 and -C3 showed improved viral cell kill. Infection following exposure to iodizing radiation appeared to produce an additive effect on cell death in most cases.

### GLV-1h153 and ^131^I combined treatment in TRAMP mice

The TRAMP mouse strain was used to evaluate the efficacy of the virus in an immunocompetent mouse model of prostate cancer. Pilot experiments showed that TRAMP mice aged between 29 and 31 weeks showed clear histological signs of either a tumor or extensive neoplasia in the prostate and associated tissues, that is, the seminal vesicles as has been described.^33^ It was, therefore, selected as the optimal time point for intervention. Animals treated with virus exhibited transient pox lesions in the skin ~7 days post treatment, indicating the replicative ability of the virus in an immunocompetent host for a prolonged period post administration. These lesions healed without intervention within ~7 days (Supplementary Figure 11). Intervention with GLV-1h153 alone extended the median survival from 50.5 days to 123.5 days, as compared with control animals (Log-Rank: *P*=0.007) (Figure 7c). The addition of ^131^I to the viral intervention further improved survival to 195 days, an additional 71.5 days over the virus alone (Log-Rank: *P*=0.0169). Comparison of the tumor growth curves show the addition of ^131^I to the viral therapy caused a significant reduction in the growth rate of the tumor (*P*=0.288).

Histological analysis revealed extensive hyperplasia of the prostate (Figure 7d) and seminal vesicles, with metastatic disease present in the lung, liver and lymph nodes (Supplementary figure 12–14). Immunohistochemical staining for the proliferation marker Ki67 confirmed prostate and seminal vesicle tissues to be highly proliferative, as well as all metastatic deposits (Supplementary figure 15–17). Immunohistochemical staining for probasin showed that probasin expression is lost as the tumor tissues become less differentiated and metastatic deposits usually did not express probasin (Supplementary figure 18–20). Normal liver tissue appears to stain positive for probasin, though this can be explained as false positive owing to the presence of probasin-related antigen in rodent liver cells.^34^ The prostate tissues treated with GLV-1h153 appear to have retained more differentiation than the control tissues and more so in the tissues treated with the combination of GLV-1h153 and ^131^I.

## Discussion

We have shown that oncolytic NIS-expressing vaccinia virus exerted significant activity against prostate cancer as a single-agent, in combination with external beam radiotherapy, and with therapeutic radioiodide. VV-NIS had potent antitumour efficacy against prostate cancer cells *in vitro*, which was most apparent at later time points, presumably as a result of ongoing viral replication (Figure 1). The presence of the NIS gene gave the virus the ability to accumulate radioiodide within infected cells and, importantly, we showed that resulted in DNA damage in uninfected bystander cells in close proximity (Figure 2). The observation that the bystander DU145 cells exhibited a greater number of γH2Ax foci could be explained by the cell cycle of the infected cells being arrested, compared with the bystander cells that are actively cycling and experiencing replication stress that would degrade single-strand breaks into double-strand breaks and thus result in more γH2Ax foci. The same effect was not seen in the other cell lines, perhaps simply because of biological variations in the rates of cell cycle or DNA repair mechanisms. This effect, which was seen in a two-dimensional *in vitro* assay, was due to emission of β-particles by ^131^I and is likely to be a significant underestimate of what would occur *in vivo* within the densely packed three-dimensional tumor environment. In combination with external-beam radiotherapy, viral oncolysis was not inhibited and the combination effectively reduced the clonogenic potential of irradiated cells (Figures 3 and 4). The mechanism of cell death involved signaling through apoptotic pathways. However, in contrast to our previous observations in melanoma cells that showed that vaccinia virus and radiation acted synergistically by modulating JNK/TNFα signaling,^32^ we did not see evidence of this effect in prostate cancer cell lines (Figure 5). This highlights the fact that different tumor types are likely to show different combinatorial effects with virus plus radiotherapy and/or chemotherapy.

We also provided *in vivo* evidence that intratumourally delivered VV-NIS was capable of driving iodide uptake in xenograft tumors and that this translated to a powerful antitumour effect. Indeed, we were able to demonstrate that the combination of VV-NIS and radioiodide was capable of delaying tumor growth significantly and of achieving long-term control in PC3 xenograft tumors (Figure 6). These data were consistent with *in vivo* evidence of gene expression—measured by GFP imaging and radioiodide uptake assay (Figure 6). To evaluate off-target viral effects, viral luciferase was used to produce bioluminescent imaging of areas of viral replication. Viral replication was high in the tumor and undetectable in other organs, indicating little or no capacity for the virus to leak from the tumor to establish infection at other sites, even in immunedeficient animals. Therefore, there is no evidence to suggest that there would be any significant off-target accumulation of iodide in any organs other than perhaps the thyroid, which as previously mentioned has not caused adverse effect in these studies. Having demonstrated this in an immunodeficient animal model, we were keen to test the therapeutic effect in immunocompetent TRAMP mice. In these experiments, we were able to confirm that intravenously delivered virus significantly extended life-span, even as a single agent. Importantly, when combined with radioiodide long-term survival was further improved, quadrupling survival compared with control animals and with 50% of the combination group surviving at the day 200 termination of the experiment (Figure 7). The ^131^I dose used in these in vivo studies is scaled down from the typical human dose and is in the range used in similar studies.^35, 36^ It is notable that those animals that received ^131^I showed no sign of late adverse effects of the treatment, indicating that any undetected off-target effects were minimal. This is reflected in clinical radiotherapy where ^131^I is used in patients with thyroid gland *in situ* on a regular basis without any long-term complications. Given that the TRAMP-C cell lines appeared relatively resistant to viral oncolysis, we hypothesize that the efficacy observed in the TRAMP model may be related to immune-mediated effects rather than direct oncolysis. The positive role of the immune system in the efficacy of oncolytic viruses has been reported in several recent studies^37, 38, 39, 40, 41^ and certainly is an avenue requiring more research, in which immunocompetent models such as the TRAMP mice and novel immune-checkpoint inhibitors will be valuable.

It had been our intention to test VV-NIS-driven radioiodide therapy combined with external-beam radiotherapy, but the efficacy of the individual component parts meant that this was not feasible. However, based on the studies reported here, we believe that viral-mediated radioiodide therapy of prostate cancer has the potential to contribute significantly to the radiation dose delivered by external-beam radiotherapy. This would theoretically improve patient outcomes (for example, progression-free and overall survival) by improving the ability of radiotherapy to secure locoregional control of prostate cancer. In addition, the selective replication-competence of VV-NIS would ensure that normal tissue toxicity would be minimized.

In summary, the data presented here not only contribute further evidence of single-agent efficacy of oncolytic vaccinia, but also point the way to further pre-clinical and clinical studies of oncolytic viruses in radio-virotherapeutic approaches. These further studies would build on the promising, but numerically limited, early-phase clinical studies utilizing adenovirus and measles which, to date, have not explored the potential additional benefit of ^131^I therapy, but have successfully demonstrated functional NIS transgene expression using non-therapeutic imaging isotopes.^18, 30^ In addition, the growing realization that oncolytic virotherapy represents a form of ‘oncolytic immunotherapy' means that further translational studies of VV-NIS, radiotherapy and other immune therapies (for example, checkpoint inhibitors) should be a priority for researchers in this field. Prostate cancer will be an excellent model system to investigate these therapies, given its accessibility for direct intratumoural injection and needle-core biopsy.

## Materials and Methods

### Cell lines

The following cell lines were used in these studies: CV1 Monkey kidney fibroblasts (ATCC, Manassas, VA, USA); Human prostate cancer cell lines PC3, DU145, LNCaP and WPMY-1 (Obtained from the laboratory of Professor Pandha, University of Surrey, UK). Murine prostate cancer cell lines TRAMP-C1 (ATCC), TRAMP-C2 (Obtained from the laboratory of Professor Vile, Mayo Clinic) and TRAMP-C3 (ATCC).

### Virus stock

GLV-1h153 (Genelux GmbH, Bernried, Germany) is an oncolytic Lister strain *Vaccinia* virus attenuated by insertion of LacZ (beta-galactosidase), hNIS and RUC-GFP (fusion gene of *Renilla* luciferase and green fluorescent protein) into the *J2R, A56R* and *F14.5 L* loci, respectively.

### Viral plaque assays

Viral titers were determined by viral plaque assay. Monolayers of CV1 cells in 24-well plates were treated with serial dilutions of the viral solution to be titred and incubated for 24 h. Cells were fixed with 2% formaldehyde (Sigma-Aldrich, Dorset, UK)/0.2% glutaraldehyde (Sigma-Aldrich) in phosphate-buffered saline (PBS, Sigma-Aldrich) and stained for 4 h with 5 mM potassium-hexa-cyanoferrat III (Sigma-Aldrich), 5 mM potassium-hexa-cyanoferrat II-tri-hydrate (Sigma-Aldrich), 2 mM magnesium chloride-hexahydrate (Sigma-Aldrich) and 0.6 mg ml^−1^ 5-Bromo-4-chloro-3-indolyl β-D-galactopyranoside (X-Gal, CalBioChem, Watford, UK) in PBS. Cells were then washed with ultrafiltered water and dried. Macroscopic X-gal-stained viral plaques were counted manually.

### MTT proliferation assay

Cell proliferation was measured by plating 1 × 10^4^ cells per well in 100 μl DMEM (Dulbecco's modified Eagle's medium, Life Technologies, Carlsbad, CA, USA) and incubating overnight before adding 100 μl virus in DMEM, diluted to the relevant MOI. At the experimental end point, 20 μl of MTT (Sigma-Aldrich) at 5 mg ml^−1^ in PBS was added. Medium was aspirated from each well after 4 h of incubation at 37 °C and crystals were solubilised in 200 μl of dimethyl sulfoxide (Fisher Scientific, Loughborough, UK). Absorbance was measured at 550 nm by a SpectraMax M5 plate reader (Molecular Devices Ltd., Wokingham, UK).

### Sulforhodamine B cytotoxicity assay

Cell viability was quantified by fixing cells with 10% trichloroacetic acid (Sigma-Aldrich) and staining with 0.05% sulforhodamine B in 1% acetic acid (Sigma-Aldrich). The sulforhodamine B bound to cells was dissolved with 10 mM TRIS and absorbance was measured at 510 nm on a SpectraMax M5 plate reader.

### Clonogenic assay

Cells were plated at 5 × 10^5^ in a T25 flask (Corning, Glendale, AZ, USA). The next day, cells were irradiated at 2 Gy and 6 h later infected with GLV-1h153 at MOI of 0.01. After 48 h of treatment, cells were washed in PBS, trypsinized and counted using a haemocytometer. Cells were then plated into six-well dishes at 400–800 cells per well. After 10–14 days, plates were stained with 0.2% crystal violet (Sigma-Aldrich) in 7% ethanol and the colonies consisting of ~50 cells or greater were counted manually.

### Caspase-3/7 luminometry assay

Relative levels of Caspase-3/7 activation 48 h post infection were measured by Caspase-Glo3/7 Assay (Promega, Southampton, UK), in which a proluminescent caspase-3/7 DEVD-aminoluciferin substrate is cleaved by active caspases 3/7, producing free aminoluciferin as a luciferase substrate. The detected levels were normalized according to the proportion of surviving cells, as determined by a tandem MTT assay carried out simultaneously.

### Western blotting

Cells were plated at 0.5 × 10^6^ in 60 mm dishes. Following various treatments, cells were harvested in ice-cold PBS, pelleted and resuspended in radioimmunoprecipitation assay buffer (50 mM Tris (pH 7.5), 150 mM NaCl, 1% NP40, 0.5% sodium deoxycholate and 0.1% SDS (all Sigma-Aldrich)) plus protease inhibitors for measurement of caspase cleavage and JNK activation. For NIS expression, cell pellets or tumors (for *in vivo* studies) were harvested and suspended in lysis buffer containing 30 mM Tris (pH 7.5), 150 mM NaCl, 5 mM CaCl_2_, 150 mM MgCl_2_, 0.5% NP40 plus protease inhibitors (Roche Diagnostics GmbH, Mannheim, Germany). Cells or tumors were then sonicated and centrifuged at 13 200 rpm per 4 °C for 20 min to remove cell debris. Protein concentration of the lysates was determined using the BCA protein assay reagent (Life Technologies). Thirty micrograms of each protein sample were resolved on sodium dodecyl sulfate–polyacrylamide gels (10–12%) and transferred to a polyvinylidene difluoride Hybond-P membrane (Amersham Biosciences, Amersham, UK). Immunodetections were performed using anti-caspase-3 (Cell Signaling, Danvers, MA, USA), anti-phosphorylated JNK (Thr 183/Tyr 185, Cell Signaling) rabbit polyclonal antibody in conjunction with a horseradish peroxidase-conjugated anti-rabbit or anti-mouse secondary antibody (GE-Healthcare, Amersham, UK). Equal loading was assessed using α-tubulin (Sigma-Aldrich) mouse monoclonal primary antibodies. The Super Signal chemiluminescent system (Life Technologies) or Immobilon Western chemiluminescent horseradish peroxidase substrate (Merck Millipore, Watford, UK) were used for detection. Quantification was performed using ImageJ software (LOCI, Madison, WI, USA).

### X-irradiation

All irradiations were done using an AGO HS MP1 X-ray unit (AGO X-Ray Ltd, Yeovil, UK) at 250 kV and at a dose rate of 0.6 Gy min^−1^, as measured directly by a PTW UNIDOS E-digital dosimeter (PTW Freiburg GmbH, Freiburg, Germany). Cells were irradiated in 24- or 96-well plates (Thermo Fisher Scientific) in single fractions up to 5 Gy.

### Chlorophenolred-β-D-galactopyranoside β-gal expression assay

Cells were treated in 96-well plates (Thermo Fisher Scientific) and at the experimental end point lysed using 0.1% Triton X-100 (Sigma-Aldrich) and 250 mM Tris pH 8.5 (Sigma-Aldrich) in water and frozen for at least 1 h at −80 °C. Upon thawing, lysates and standards were mixed 1:1 with chlorophenolred-β-D-galactopyranoside-staining solution (60 mM disodium phosphate, 1 mM magnesium sulfate, 10 mM potassium chloride, 50 mM 2-mercaptoethanol, 1 mg ml^−1^ chlorophenolred-β-D-galactopyranoside (all Sigma-Aldrich)) and incubated at 37 °C until a gradient was visible in the standards (at ~2 h). Absorbance was then measured at 578 nm in a SpectraMax M5 plate reader. Sample β-gal concentrations were interpolated from the standards by non-linear regression in GraphPad Prism v5 (GraphPad Software, La Jolla, CA, USA).

### NIS mRNA quantitative reverse transcriptase PCR assay

Cells were treated in 24-well plates for 24 h before lysis and collection of RNA using RNeasy mini kit (Qiagen, Manchester, UK). Complementary DNA was prepared using a SensiFAST cDNA synthesis kit (Bioline Reagents Limited, London, UK) and qPCR performed using an SLC5A5 (NIS gene)-specific TaqMan gene expression assay kit (Applied Biosystems, Warrington, UK) and run on a StepOne Plus thermal cycler (Applied Biosystems).

### Radioiodide uptake assay

Cells were plated in 24-well plates (Nunc) at 1 × 10^5^ cells per well in 500 μl DMEM and incubated overnight before infection with virus. At the experimental end point, 1 μCi (0.37 MBq) of ^131^I (Perkin Elmer, Waltham, MA, USA) was added to each well for 1 h. Duplicate wells received ^131^I pre-mixed with 50 μM potassium perchlorate (Sigma-Aldrich). All wells were washed with copious amounts of PBS before detaching cells with 100 μl 1 m sodium hydroxide (Sigma-Aldrich) for 20 min. Gamma emissions from each sample were measured with a Wallac 1470 Wizard automatic gamma counter (Perkin Elmer).

### Confocal microscopy

Cells were seeded into glass bottomed 35 mm dishes (MatTek Corporation, Ashland, MA, USA), and infected at an MOI of 0.01 24 h later. Following a 24 h infection period cells were then irradiated or the media was spiked with 5 μCi (1.85 MBq) of ^131^I. Cells were then incubated for 1 h to allow H2Ax foci to form at the site of any DNA double-strand breaks induced, before being fixed in 4% paraformaldehyde. Permeabilised with 0.2% Triton X-100 in PBS for 20 min. H2aX foci were detected by a 1:200 dilution of rabbit anti-γH2aX primary antibody (Cell signaling) incubated at 4 °C overnight and Alexafluor-546 conjugated secondary antibody (Life Technologies) diluted 1:1000 at room temperature for 90 min. DNA was stained with 1:10 000 dilution of DAPI (Life technologies). Quantification of the confocal images was done using CellProfiler 2.0 (Broad Institute, Cambridge, MA, USA) software configured to recognize cell nuclei, GFP-expressing cells and γH2Ax foci. The bystander cells were classified as cell nuclei lying wholly or partially within a 20 μm perimeter of GFP-positive cells.

### *In vivo* xenograft studies

Xenograft tumors (PC3) were established by injecting 3 × 10^6^ cells in 100 μl PBS subcutaneously in to the right flank of CD1 nude mice (Charles River Ltd., Harlow, UK). Tumors were allowed to grow to 5–8 mm in diameter and randomly allocated to treatment groups before beginning therapy. All animal work was carried out in compliance with UK Home Office regulations and under the scrutiny of the Institutional Review Board.

Virus at the relevant concentration was administered intratumourally in 100 μl PBS. Control animals received intratumoural injections of PBS alone. A single cutaneous puncture site and multiple intratumoural injection tracks were used to improve the distribution of injectate within the tumor. After 48 h, ^131^I was administered by intraperitoneal injection (1 mCi (37 MBq) in 100 μl Hanks balanced salt solution.

Prior to administration of ^131^I, cage water was supplemented with 5% Lugol's iodine solution (Sigma-Aldrich) for a minimum of 72 h to attempt to saturate the thyroid gland with non-radioactive iodide. This was replaced with normal drinking water 24 h before ^131^I administration.

Prior to tumor irradiation, mice received intraperitoneal injections of 100 μl of a 1:1:4 solution of Hypnorm (0.315 mg ml^−1^ fentanyl citrate and 10 mg ml^−1^ fluanisone; Janssen Pharmaceutica, Raritan, NJ, USA), Hypnovel (5 mg ml^−1^ midazolam; Roche Products Ltd., Welyn Garden City, UK) and sterile water.

Tumors were measured twice weekly in two dimensions using Vernier callipers and the volume was estimated using the formula: (width × length^2^)/2.

For some experiments, tumor iodide uptake was measured by excision of the whole tumor and other tissues, and detection of gamma emissions using a Wallac 1470 Wizard automatic gamma counter (Perkin Elmer). Results were normalized to the weight of the tumor/thyroid gland. GFP imaging was performed under inhalational anesthesia (Vetflourane, Virbac Animal Health, Bury St Edmunds, UK) with IVIS Lumina II imaging system (Caliper Life Sciences, Waltham, MA, USA). GFP fluorescence was detected using an excitation wavelength of 465 nm, a GFP-specific emission filter and an exposure time of 0.2 s. Bioluminescence was measured following intraperitoneal administration of 100 μg h-Coelenterazine-SOL (Nanolight Technologies, Pinetop, AZ, USA) immediately prior to imaging.

### TRAMP mouse *in vivo* studies

The TRAMP mouse model was acquired from the Jackson Laboratory (Bar Harbor, ME, USA). A breeding colony was established in the C57BL/6 background to produce heterozygous TRAMP males. Characterization studies were carried out to observe the rate of tumor growth and select a time point for intervention based on the level of disease progression. Animals were culled at a range of time points and the prostate gland and seminal vesicles were dissected and observed histologically. Animals were found to develop prostate adenocarcinoma, as well as seminal vesicle epithelial stromal tumors that resemble phyllodes tumors of the human breast, as has been previously described in this model.^33^

Therapeutic experiments were carried out on heterozygous TRAMP males aged 29–31 weeks. Animals received 5 × 10^7^ PFU of GLV-1h153 in 100 μl PBS by tail vein injection. Five days later, animals received 1 mCi ^131^I in 100 μl Hanks balanced salt solution by intraperitoneal injection. All groups were given Lugol's iodine supplement as described previously. The animals were observed long-term for disease progression and culled if showing signs of discomfort. Animals with abdominal tumors not causing any obvious discomfort were culled if the tumor was palpable and over 1 cm in diameter. Post mortem dissection of all mice was done to enable histological analysis of the extent of the tumor progression and metastasis. Tissues were paraffin embedded and sectioned before Shandon Harris Hematoxylin (Thermo Fisher Scientific) and eosin (Leica Biosystems, Wetzlar, Germany) staining, and immunohistochemical staining with Ki67 (Dako UK Ltd., Ely, UK) and probasin (Santa-Cruz Biotechnology Inc., Dallas, TX, USA) antibodies with Shandon Gill Hematoxylin counterstain (Thermo Fisher Scientific).

### Statistical analysis

All statistical tests including *t*-tests, one-way ANOVA, two-way ANOVA with Bonferroni post-tests, Kaplan–Meier survival analysis and associated Log-Rank tests were performed using GraphPad Prism version 5 (GraphPad Software).
