# Supplementary figures and images for: Oncolytic vaccinia virus as a vector for therapeutic sodium iodide symporter gene therapy in prostate cancer
Source: Gene Ther. 2016 Feb 18;23(4):357–68. doi: 10.1038/gt.2016.5 (PMC4827015; doi:10.1038/gt.2016.5)

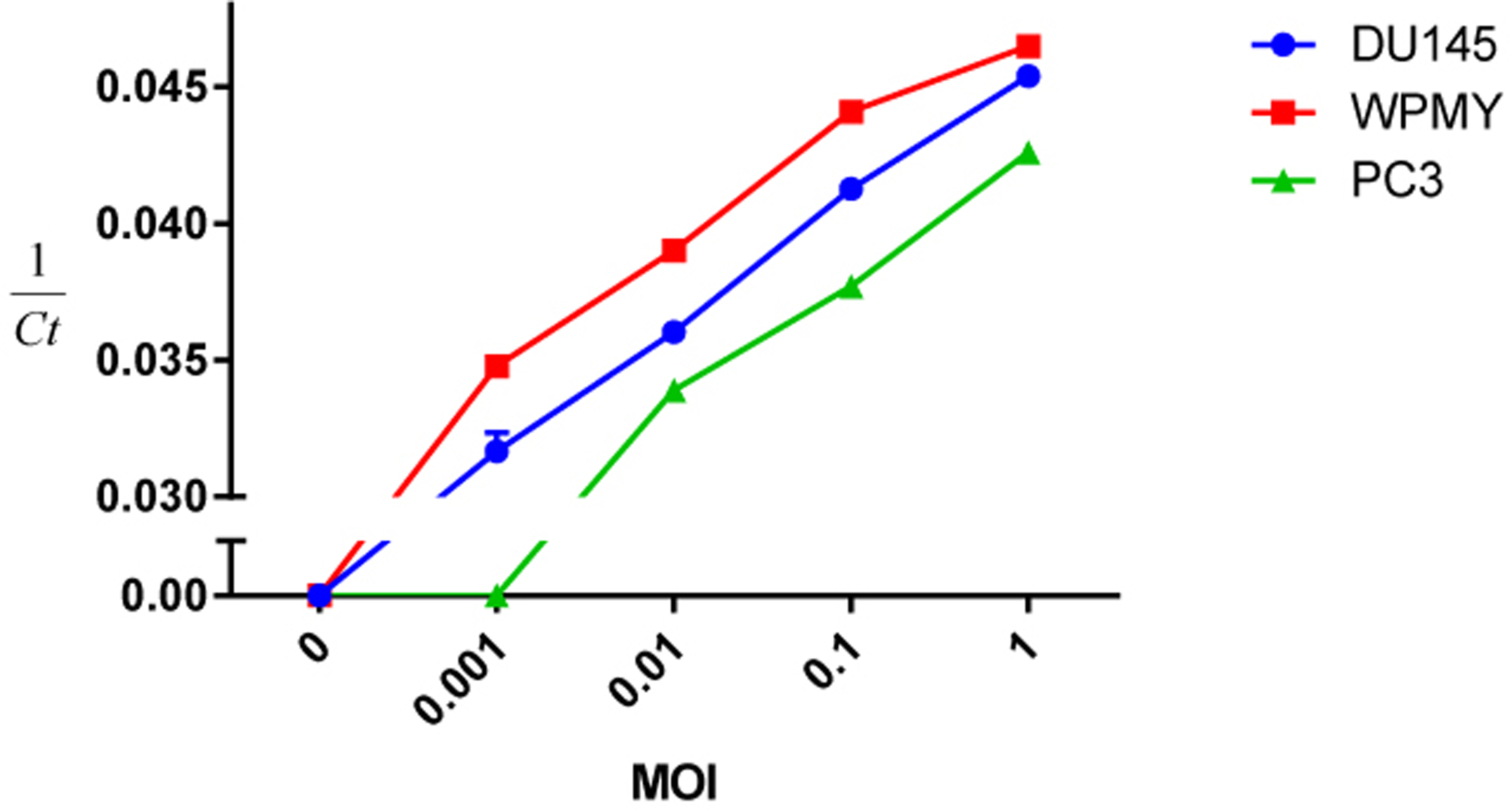

Supplement: Supplementary Figure 1 [file gt20165x1.tif]

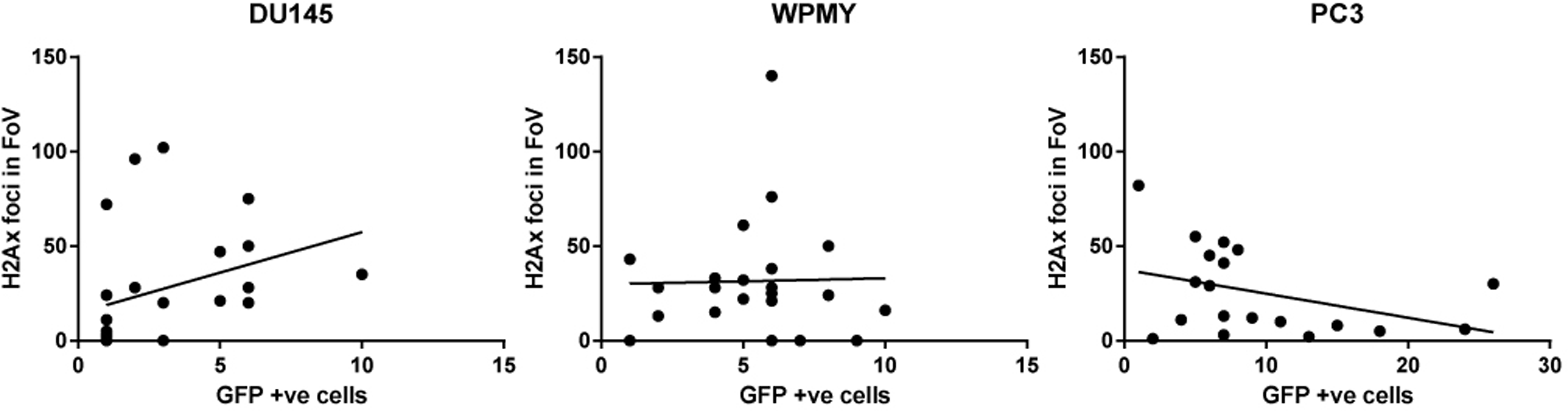

Supplement: Supplementary Figure 2 [file gt20165x2.tif]

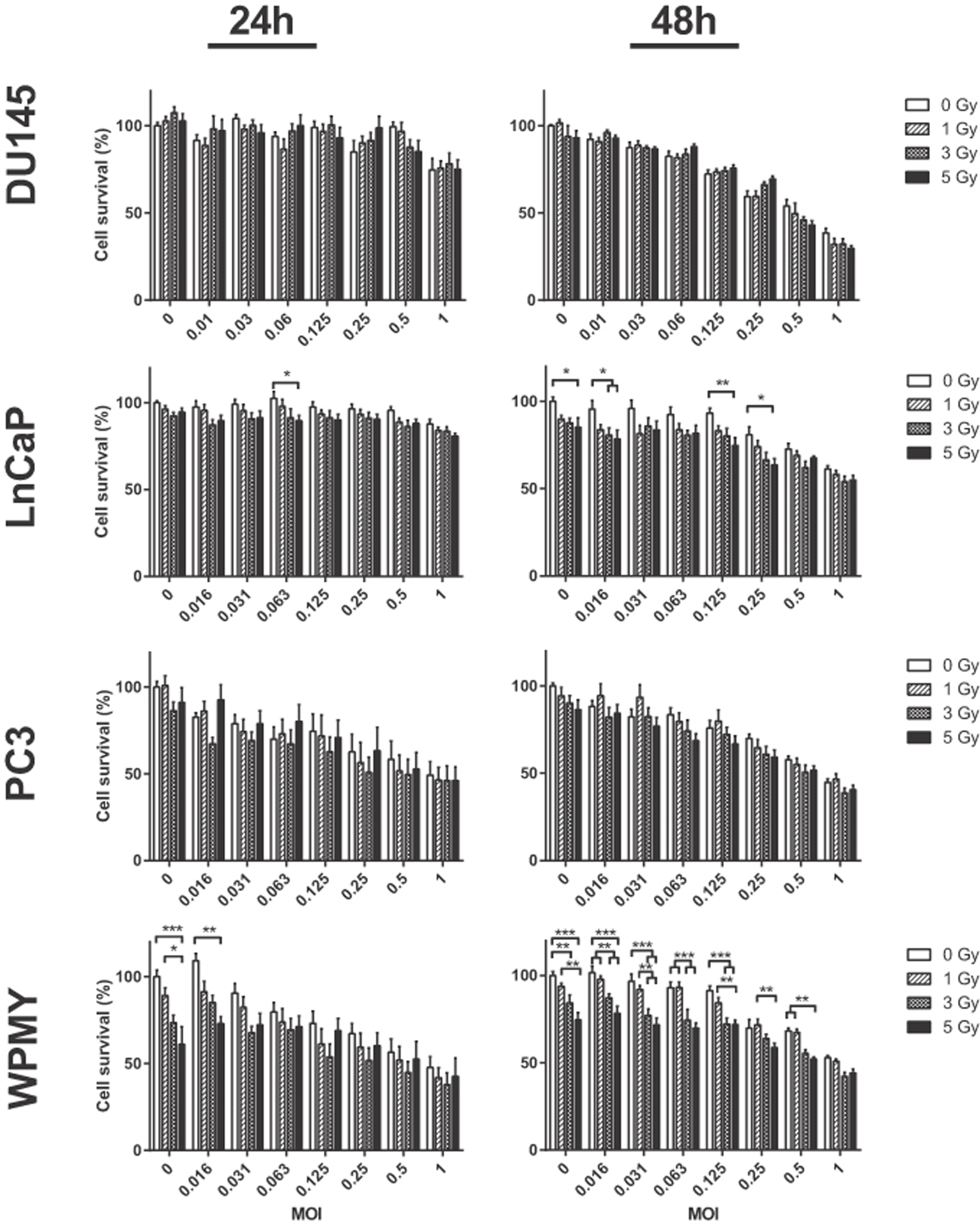

Supplement: Supplementary Figure 3 [file gt20165x3.tif]

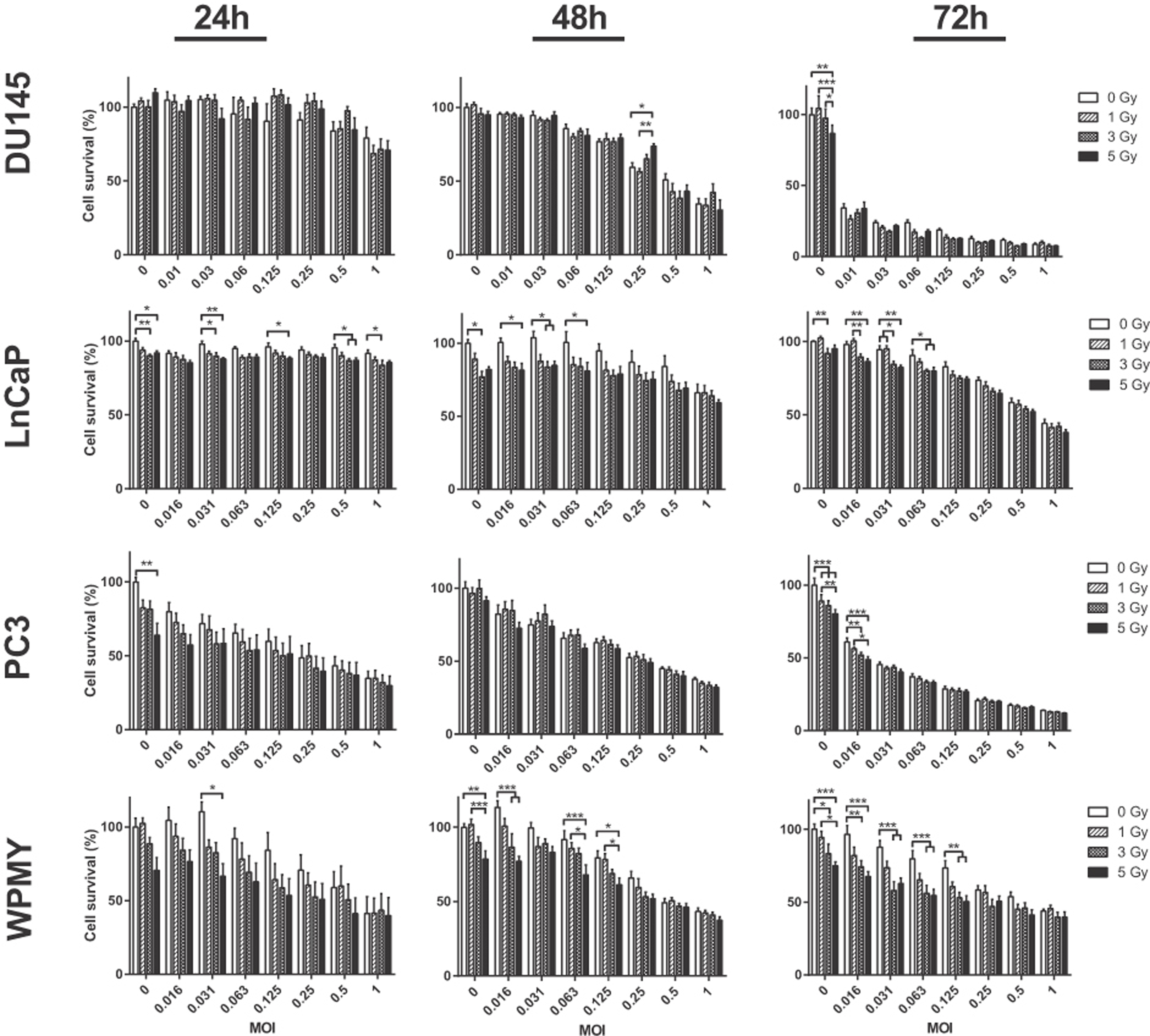

Supplement: Supplementary Figure 4 [file gt20165x4.tif]

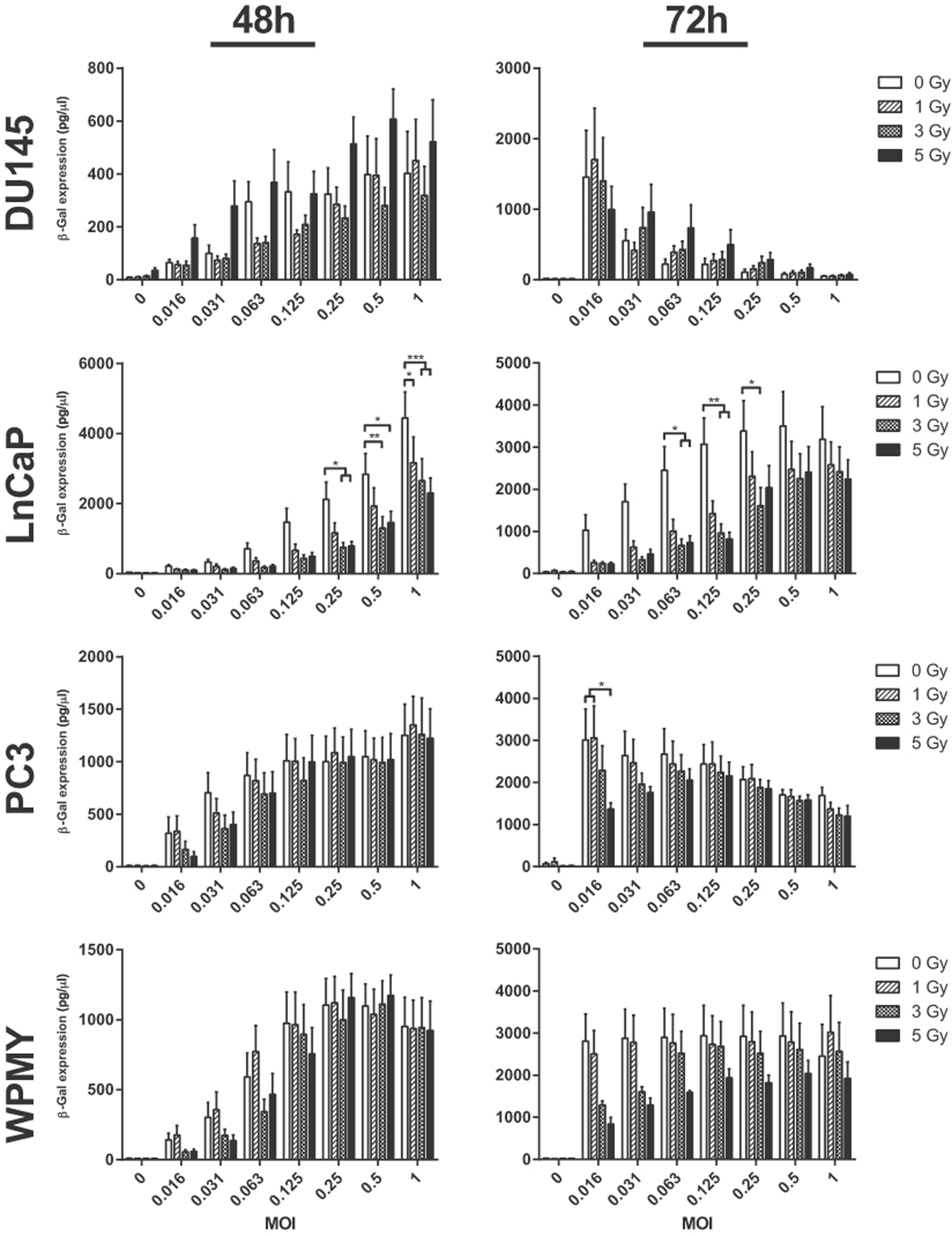

Supplement: Supplementary Figure 5 [file gt20165x5.tif]

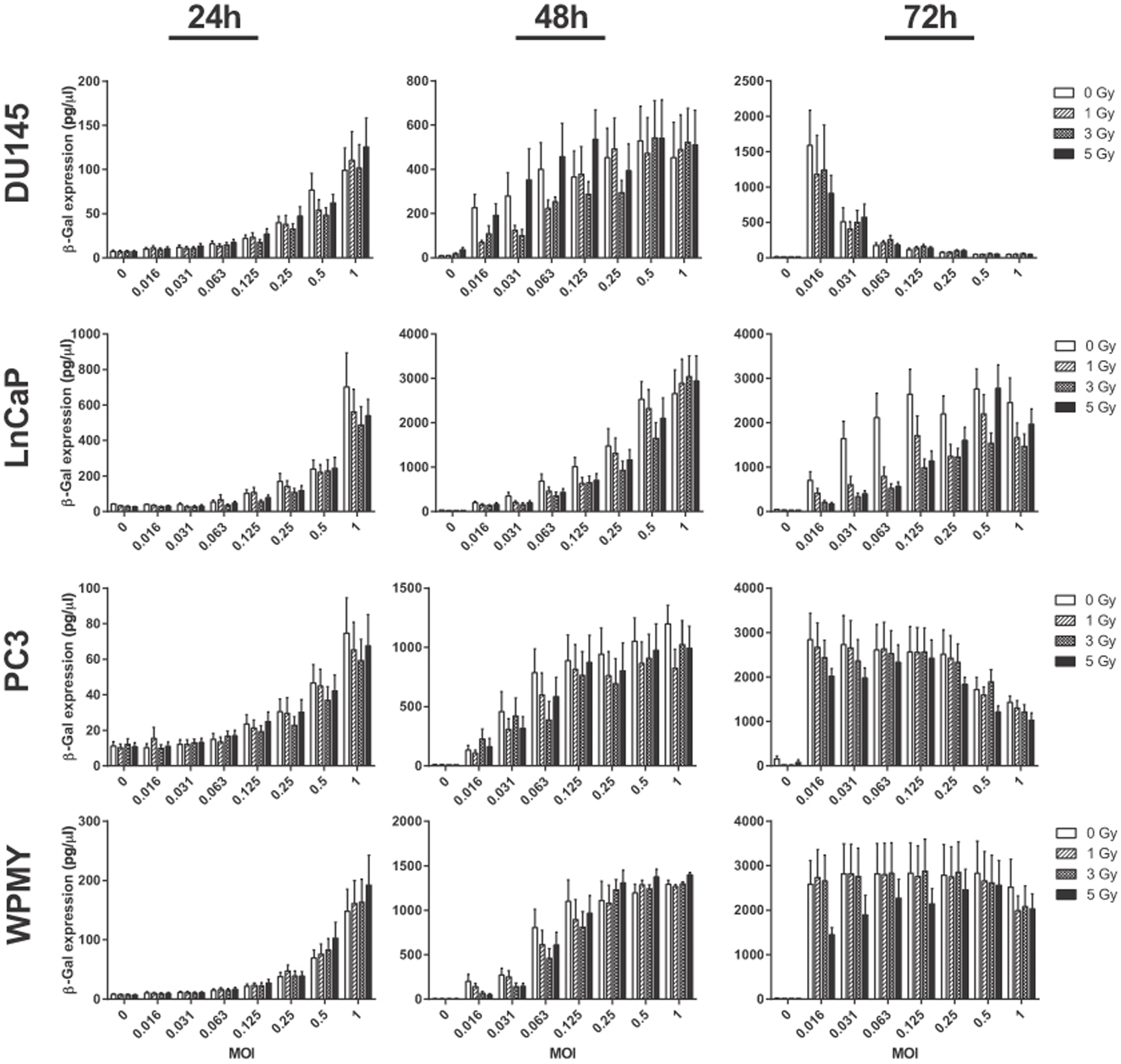

Supplement: Supplementary Figure 6 [file gt20165x6.tif]

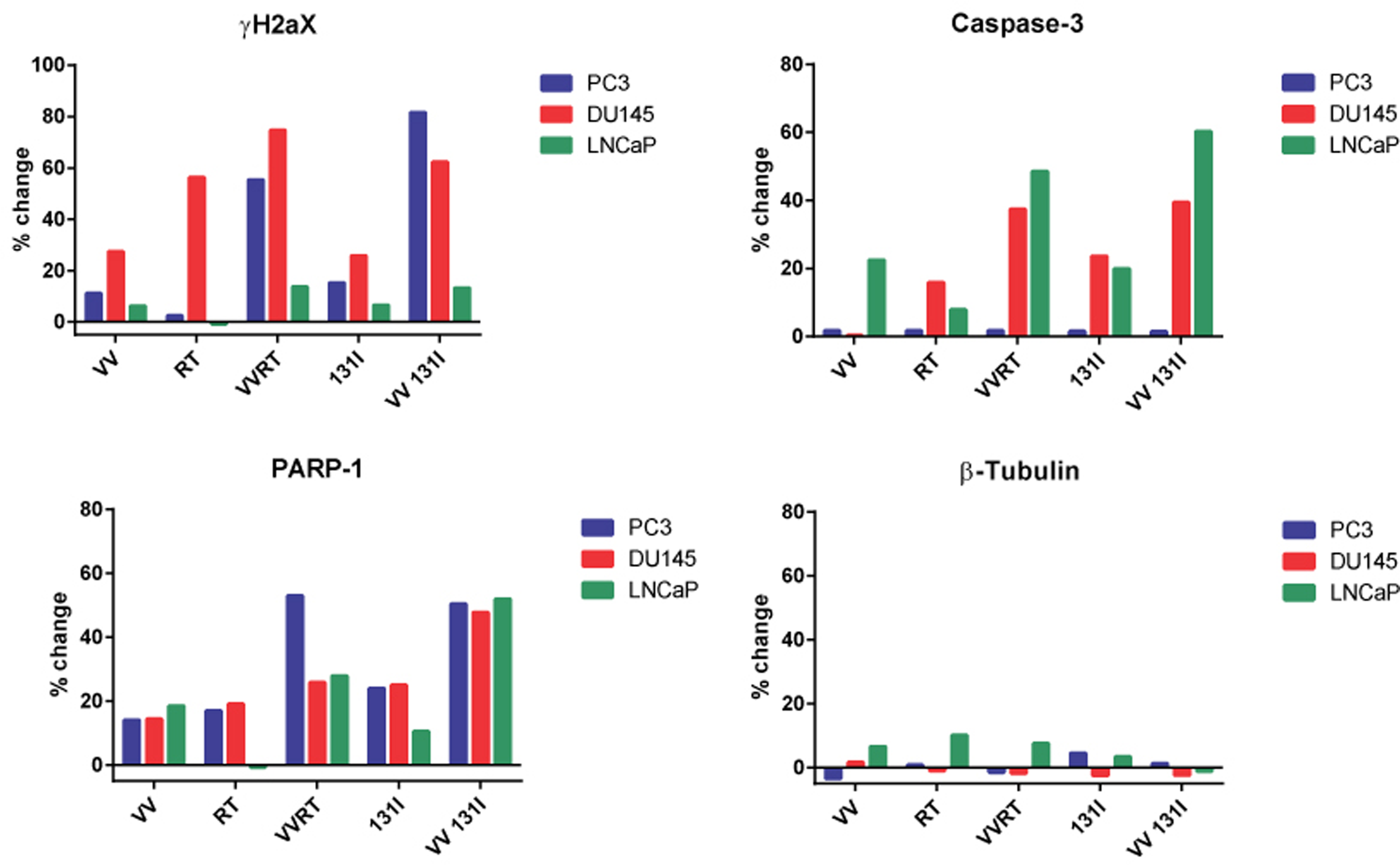

Supplement: Supplementary Figure 7 [file gt20165x7.tif]

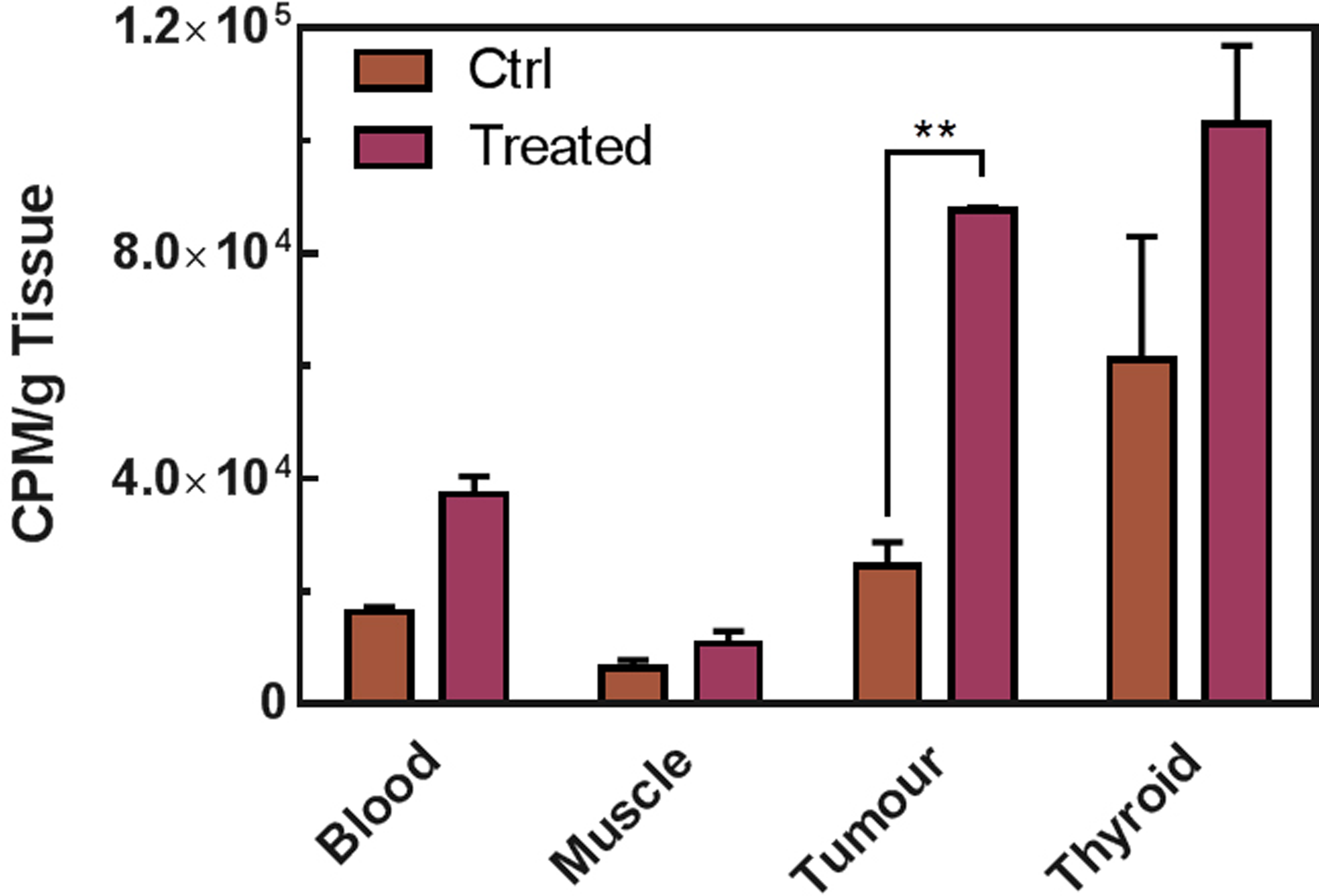

Supplement: Supplementary Figure 8 [file gt20165x8.tif]

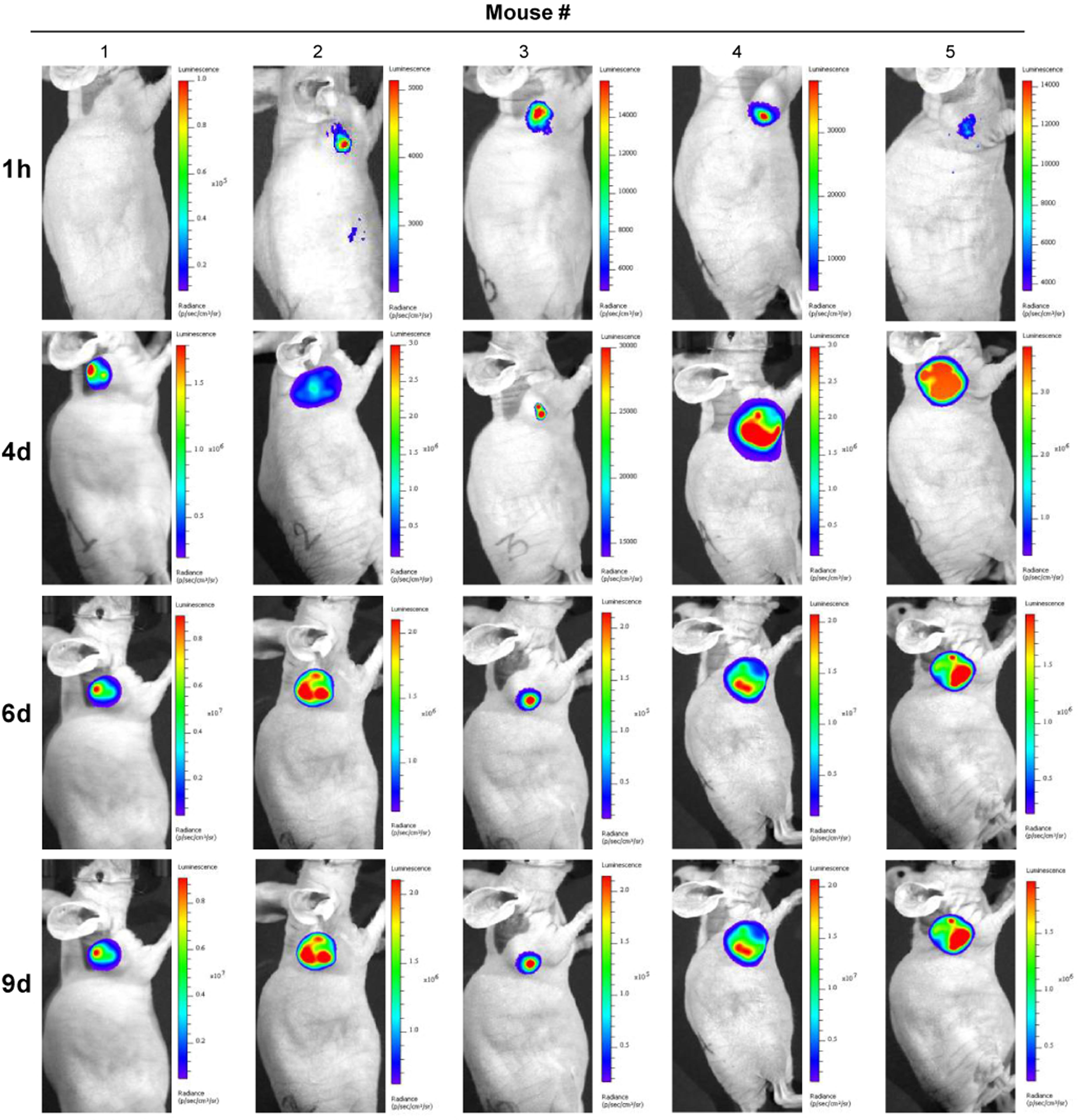

Supplement: Supplementary Figure 9 [file gt20165x9.tif]

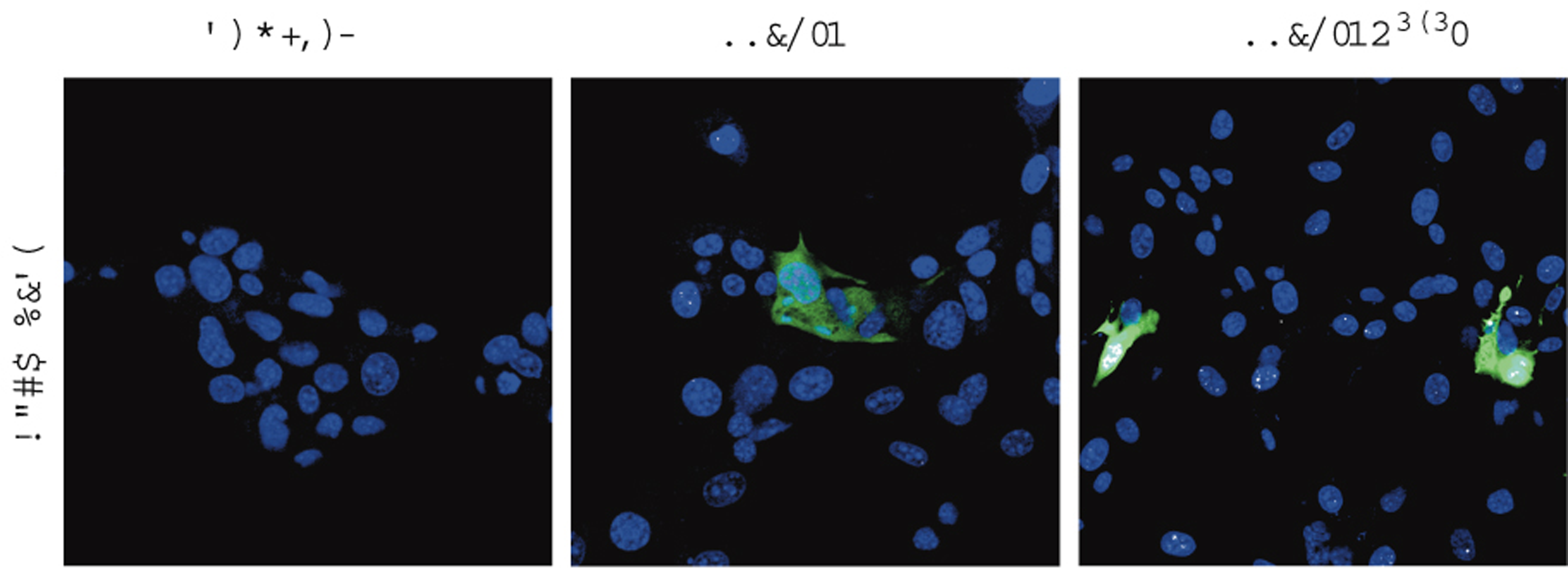

Supplement: Supplementary Figure 10 [file gt20165x10.tif]

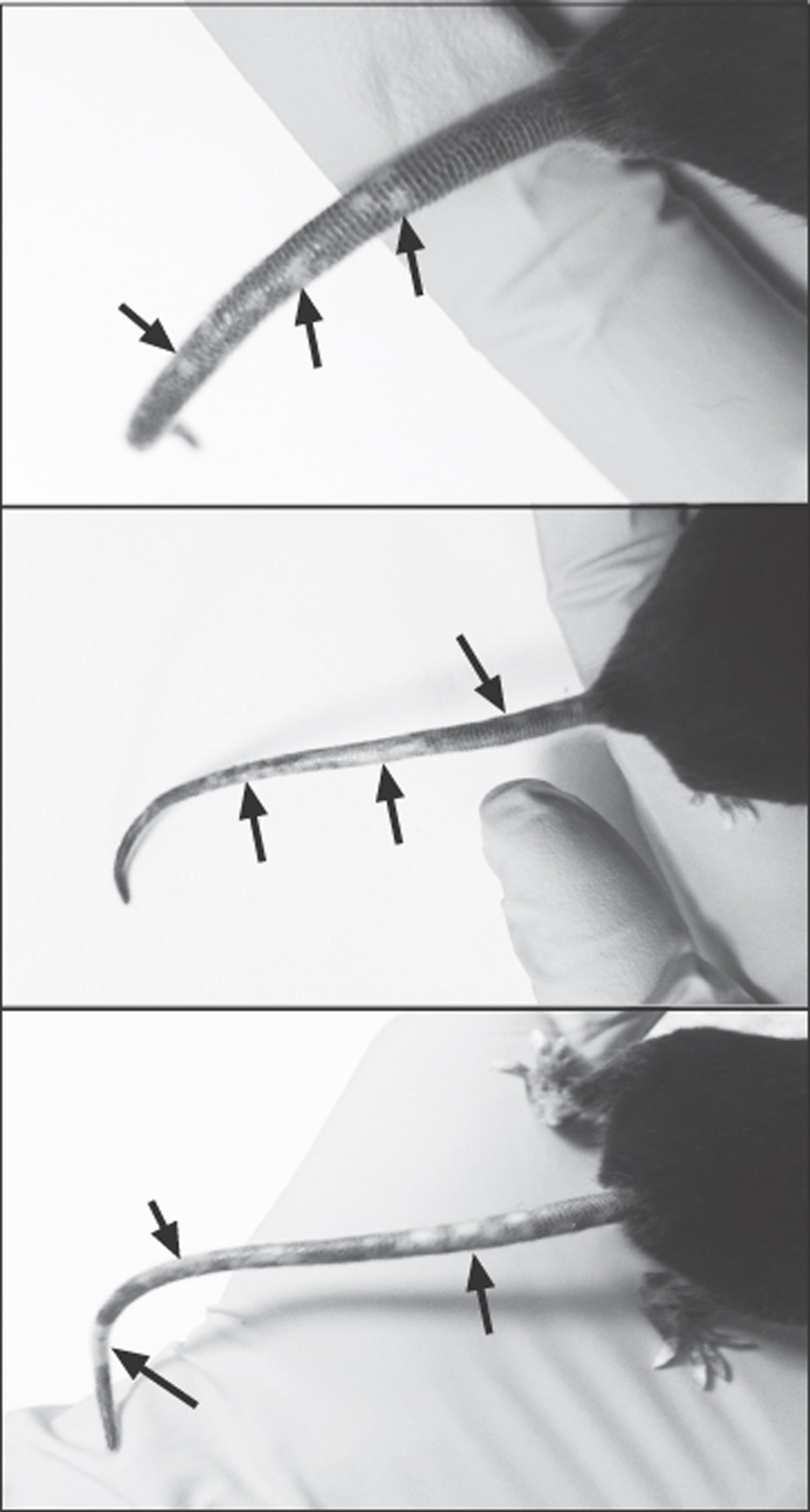

Supplement: Supplementary Figure 11 [file gt20165x11.tif]

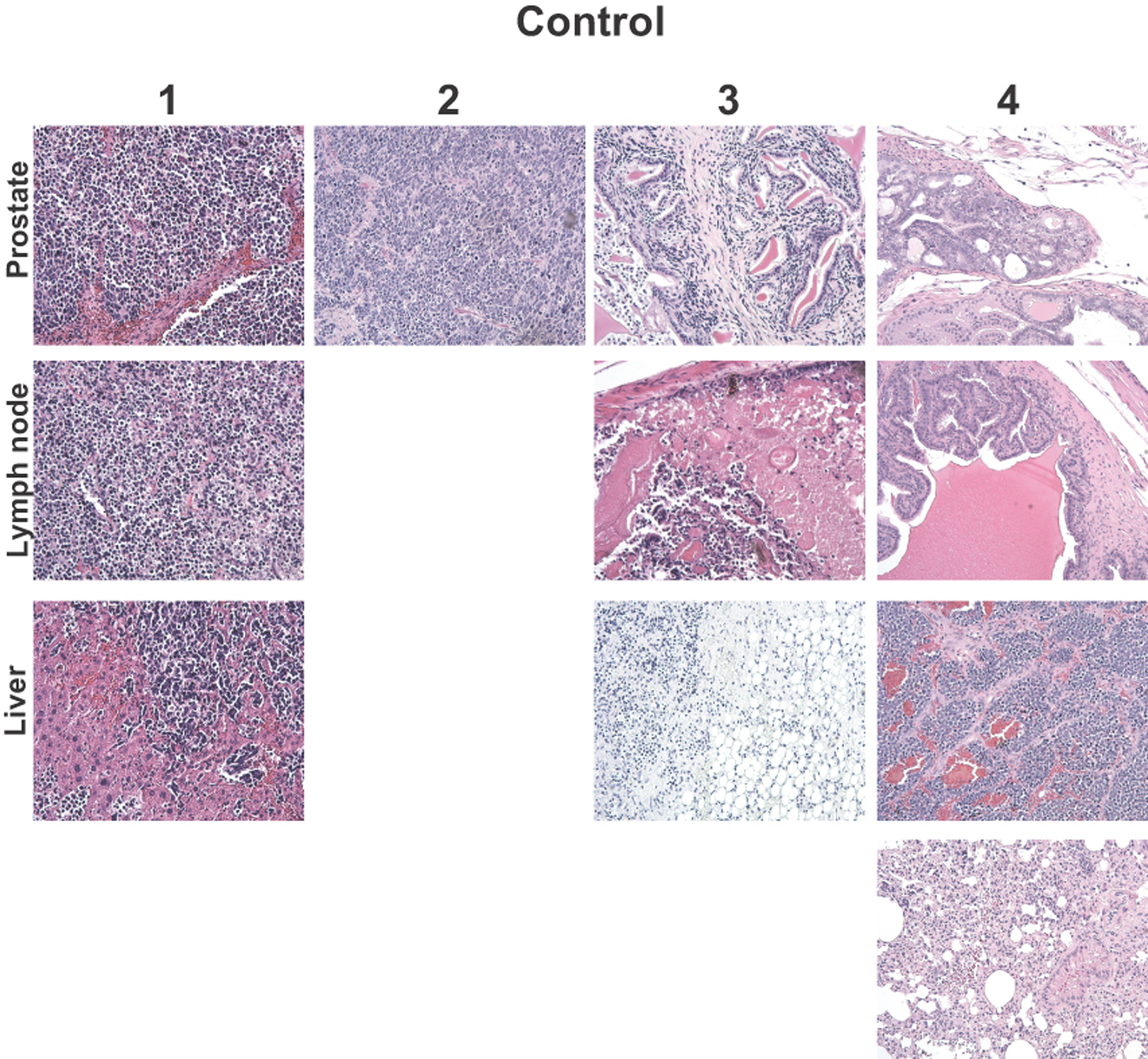

Supplement: Supplementary Figure 12 [file gt20165x12.tif]

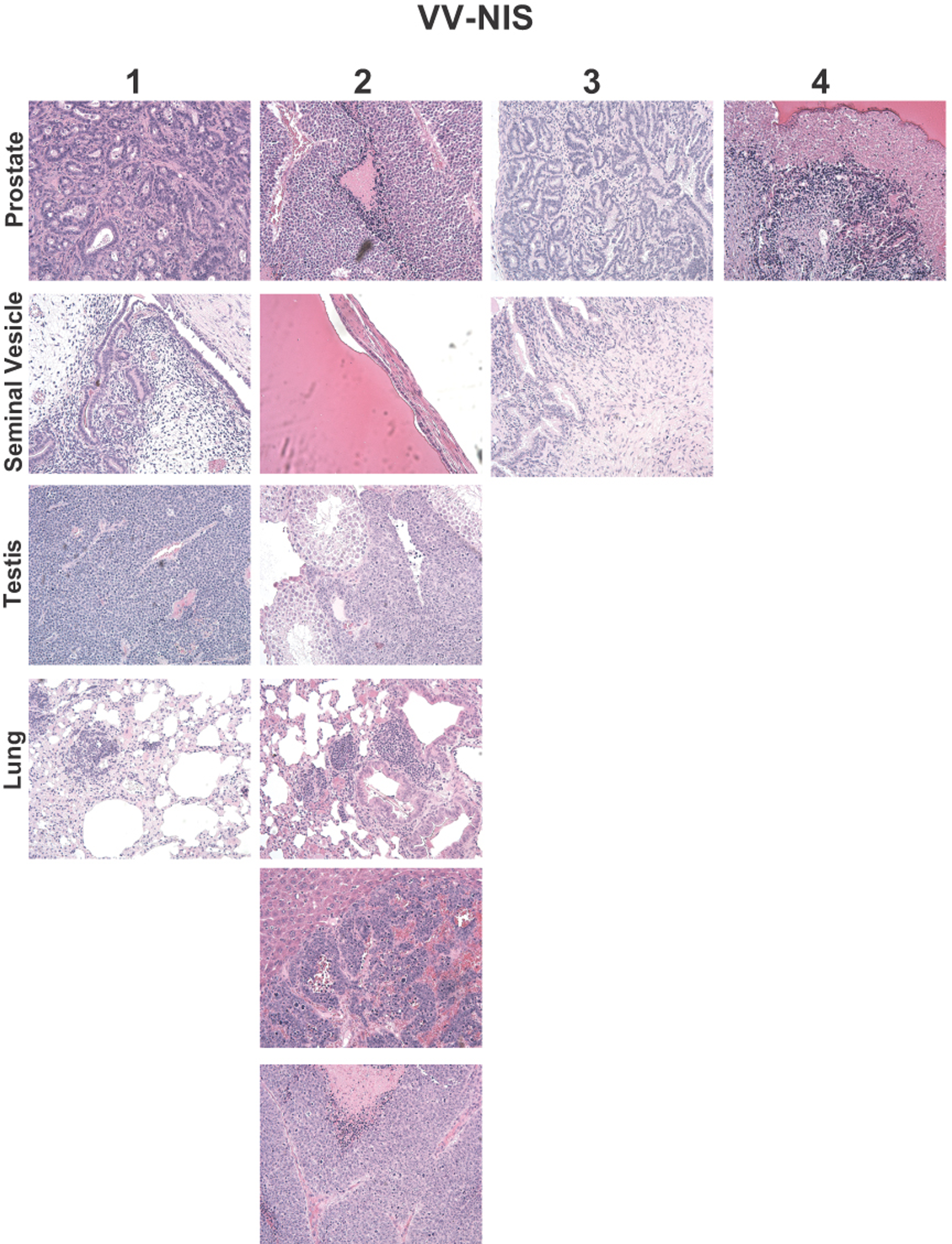

Supplement: Supplementary Figure 13 [file gt20165x13.tif]

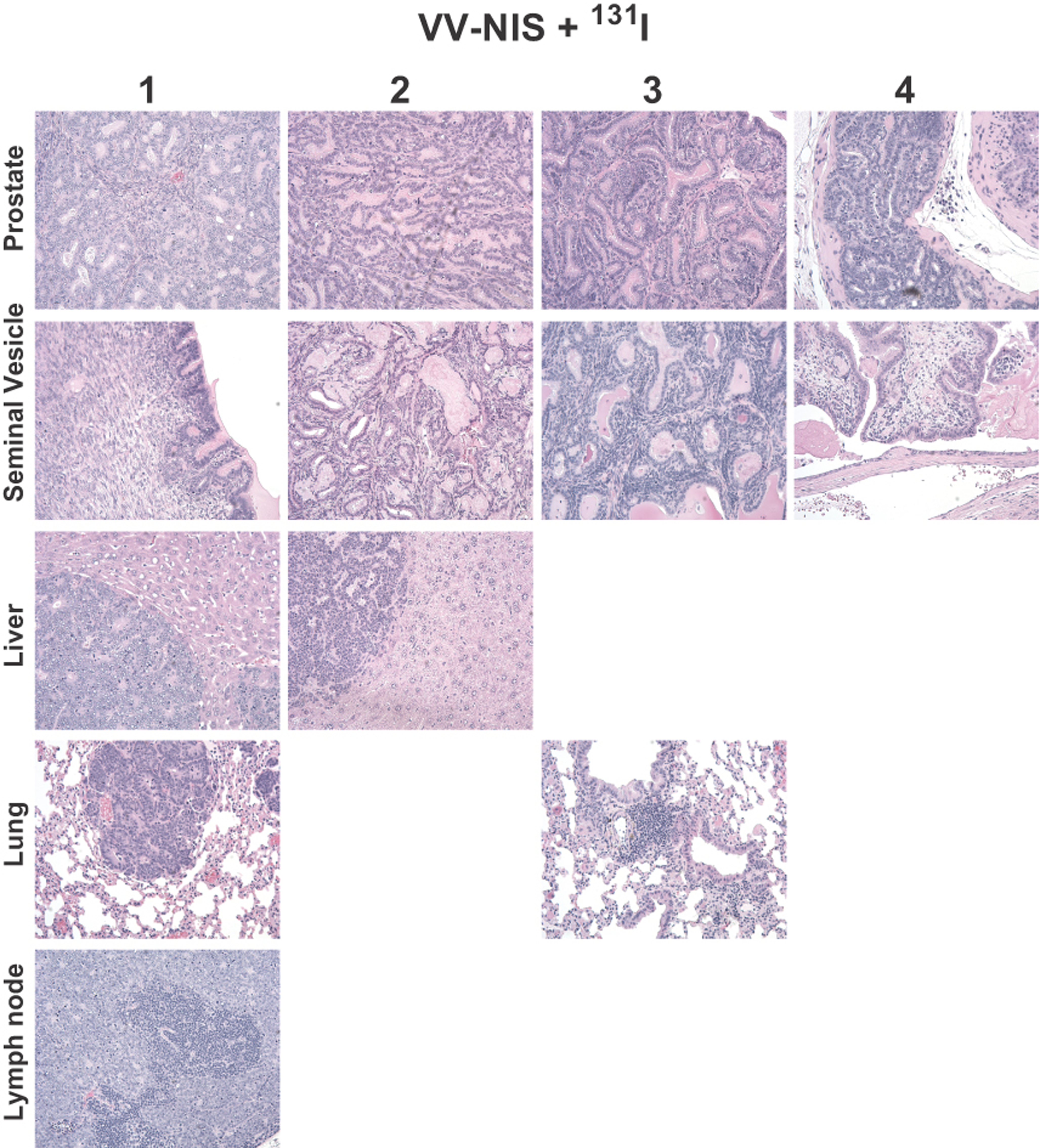

Supplement: Supplementary Figure 14 [file gt20165x14.tif]

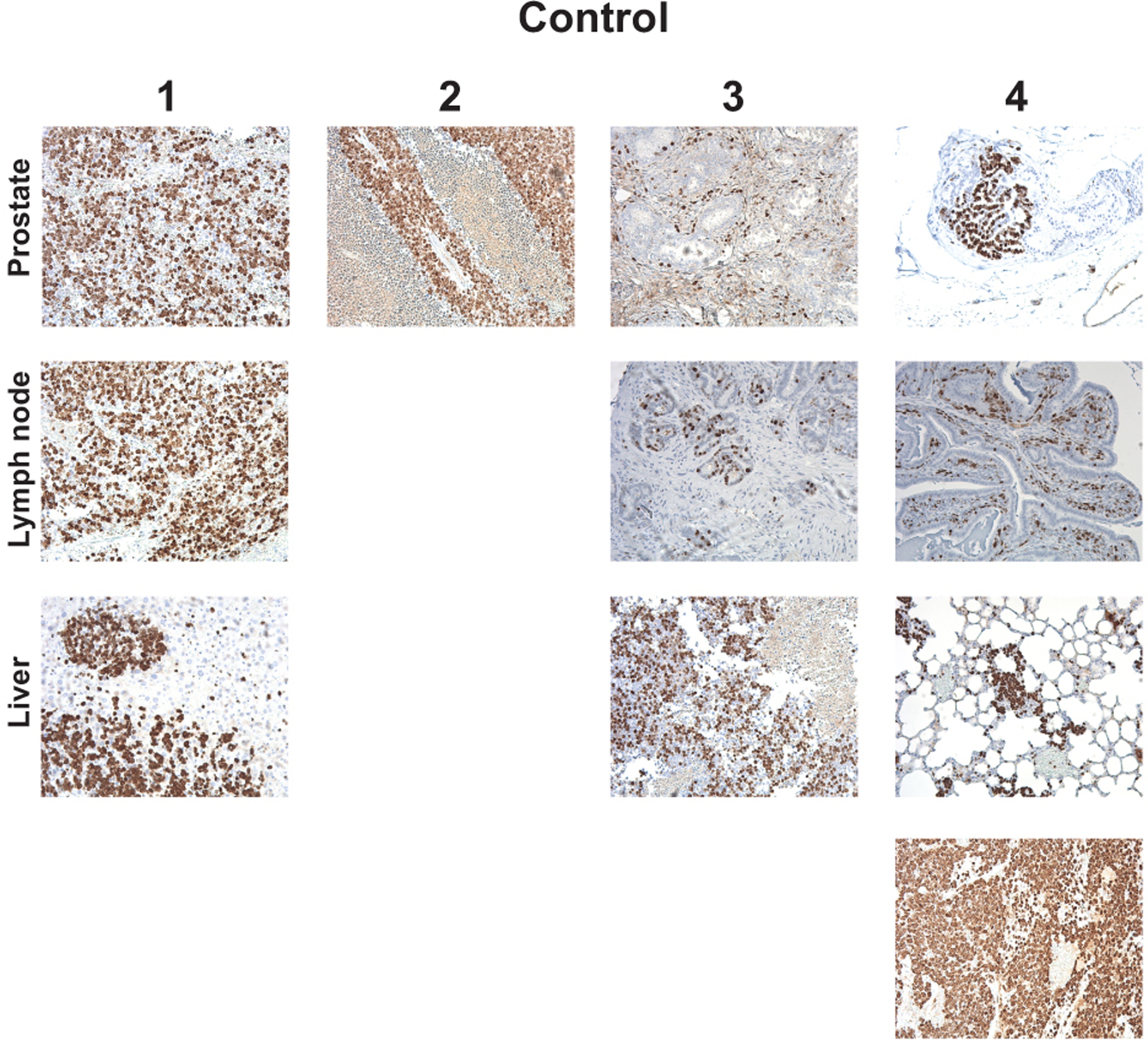

Supplement: Supplementary Figure 15 [file gt20165x15.tif]

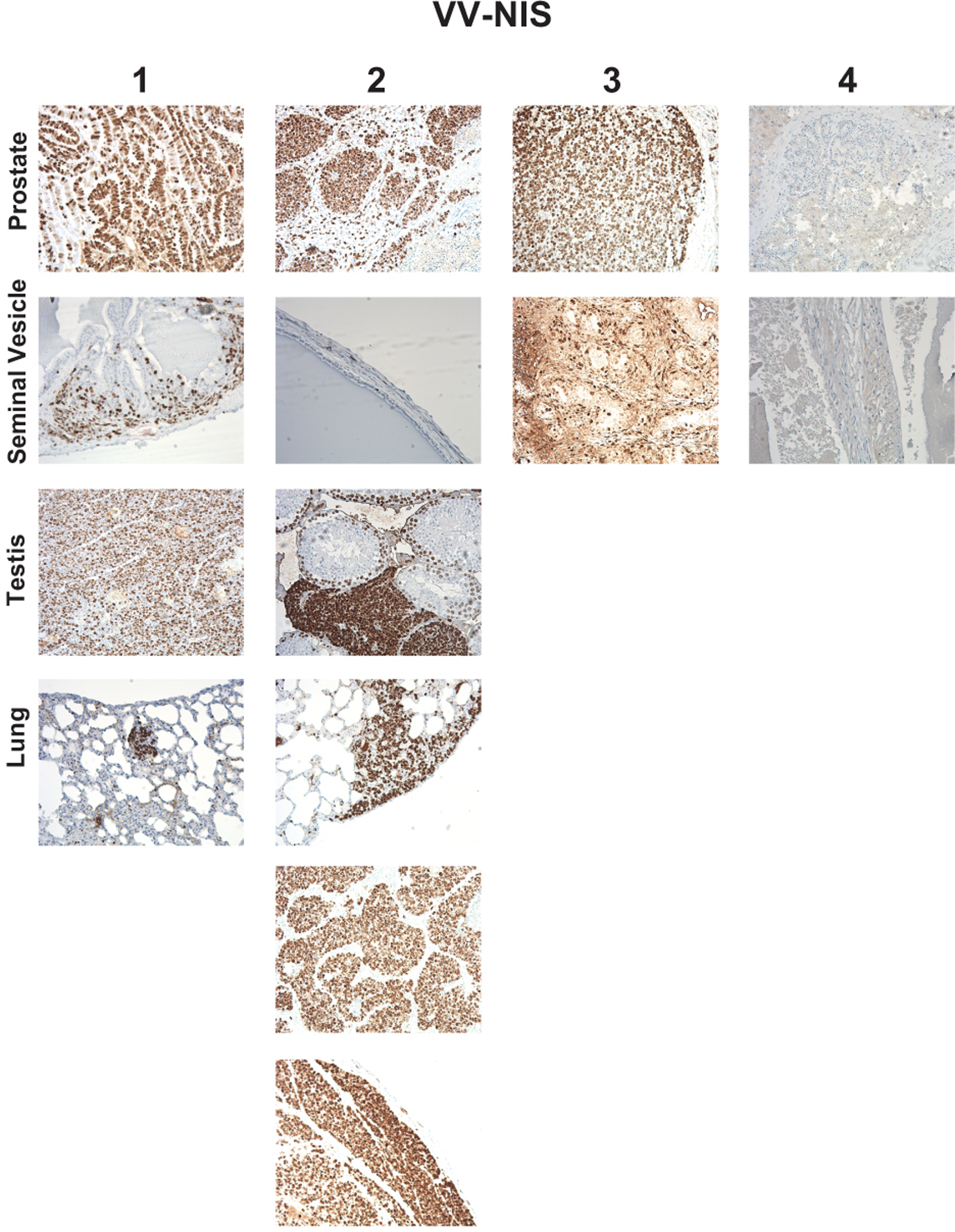

Supplement: Supplementary Figure 16 [file gt20165x16.tif]

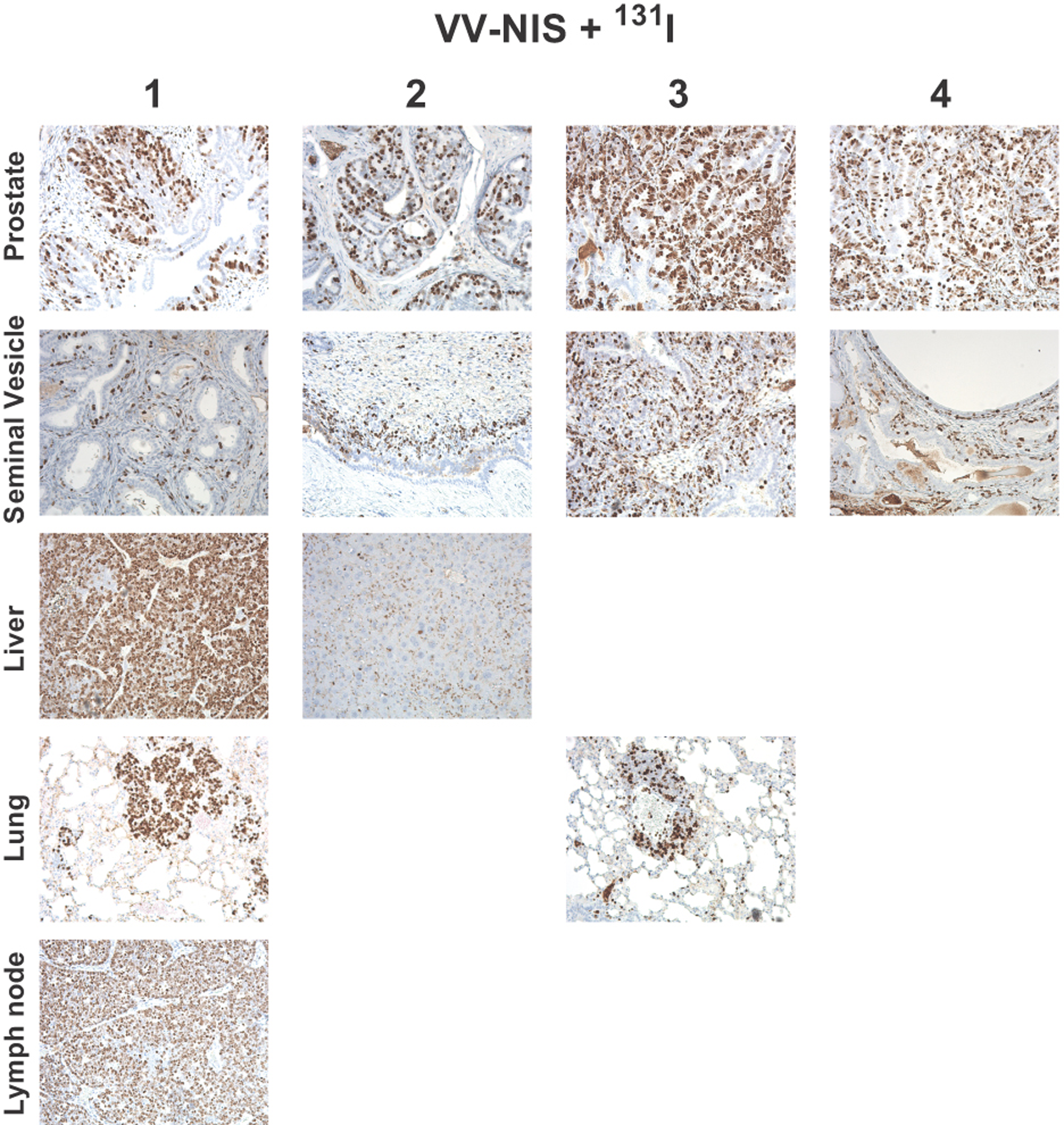

Supplement: Supplementary Figure 17 [file gt20165x17.tif]

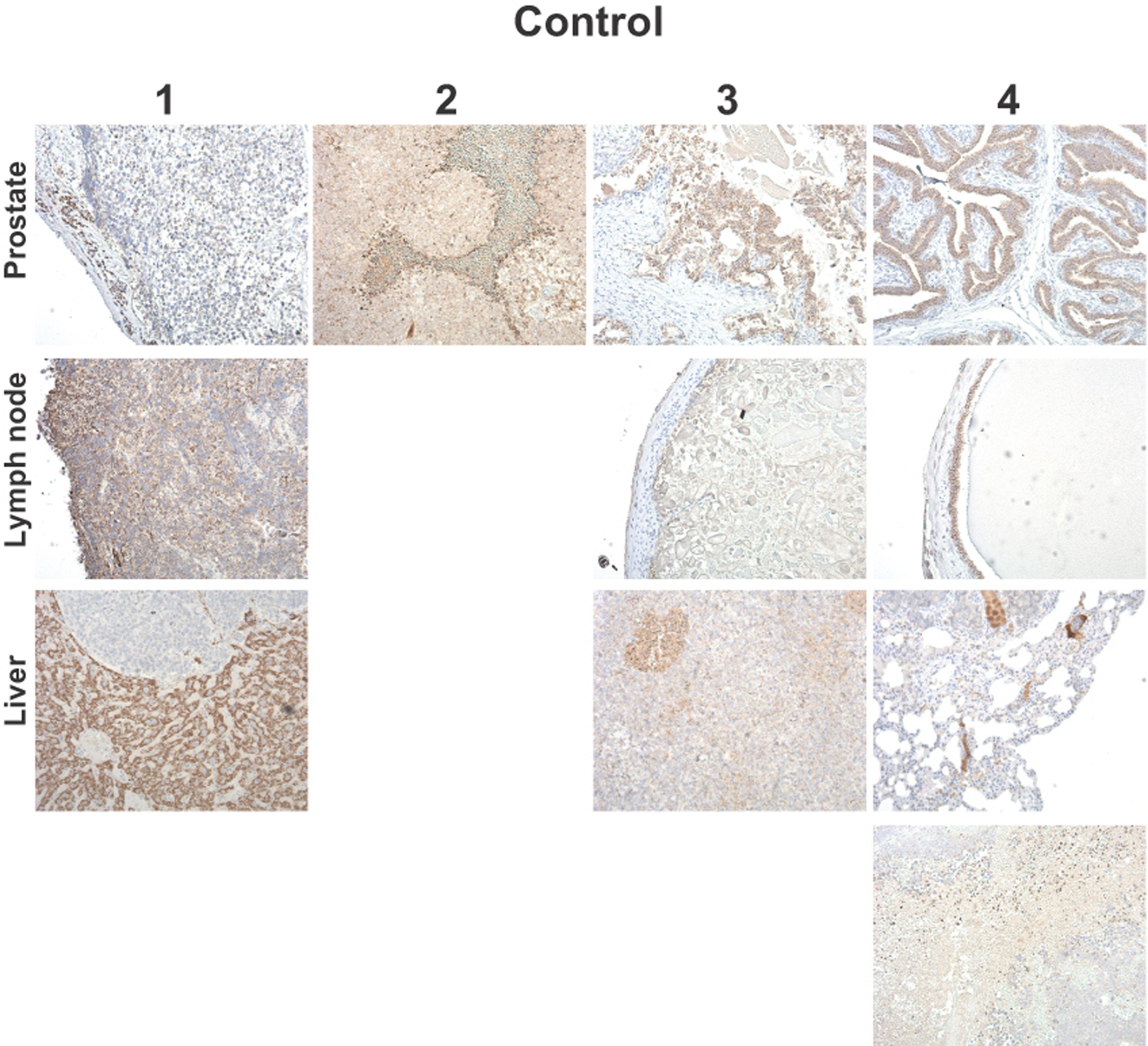

Supplement: Supplementary Figure 18 [file gt20165x18.tif]

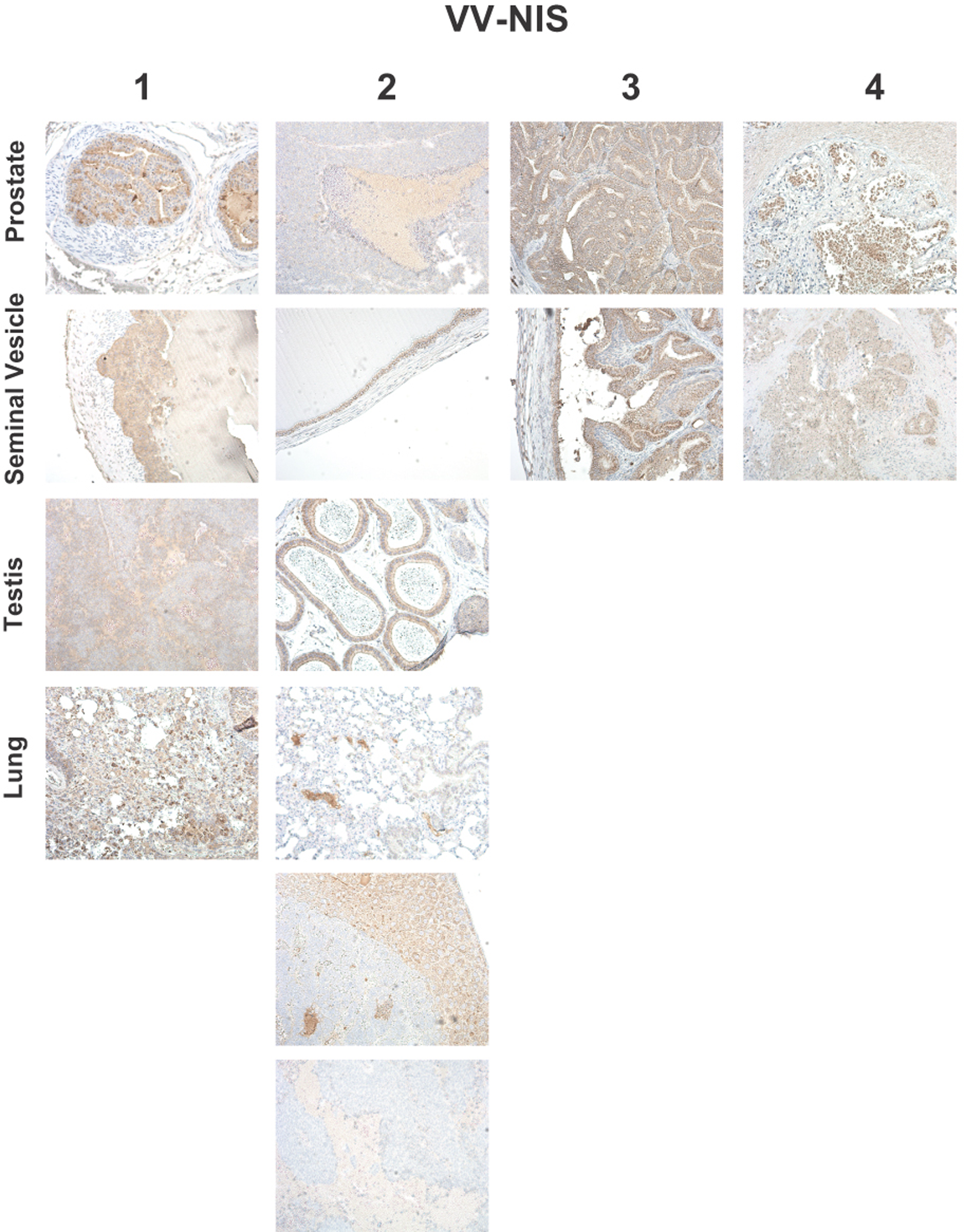

Supplement: Supplementary Figure 19 [file gt20165x19.tif]

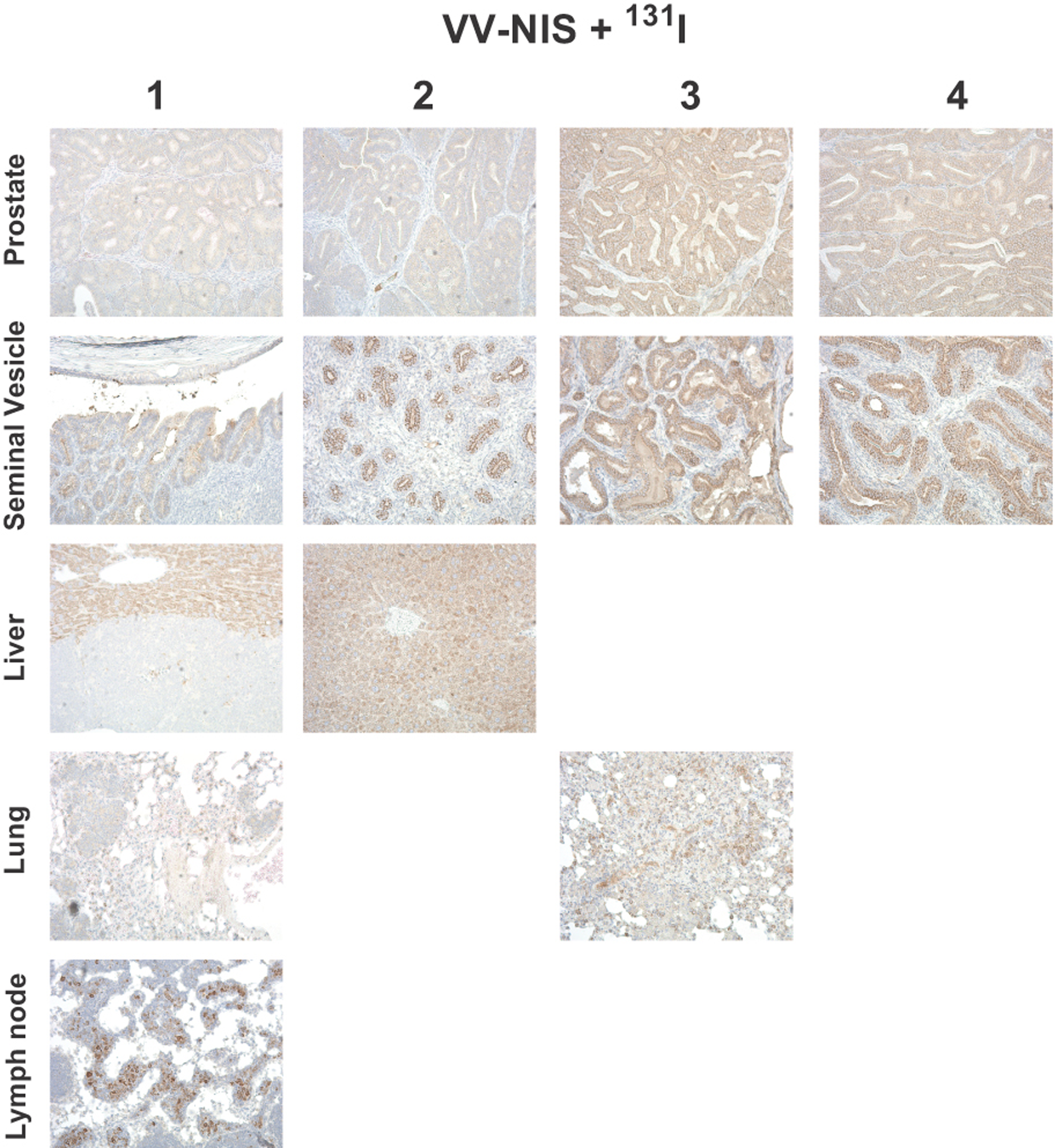

Supplement: Supplementary Figure 20 [file gt20165x20.tif]
