# Supplementary material for: Oncolytic vaccinia virus as a vector for therapeutic sodium iodide symporter gene therapy in prostate cancer
Source: Gene Ther. 2016 Feb 18;23(4):357–68. doi: 10.1038/gt.2016.5 (PMC4827015; doi:10.1038/gt.2016.5)
Supplement: Supplementary Figure Legends [file gt20165x21.docx]

Supplementary Figure 1

Detection of NIS mRNA in prostate cells infected with virus for 24 hours by qRT-PCR.

Supplementary Figure 2

An alternative analysis of by-stander effect by correlation of the quantified H2Ax foci and infected cells using the data presented in Figure 2B.

Supplementary Figure 3

Prostate cancer cells treated with GLV-1h153 following treatment with external beam radiation. Reduction of proliferative capability was measured by MTT assay at 24 and 48 hours. Standard errors of the mean are shown. Significance is the result of 2-Way ANOVA with Bonferroni multiple comparisons test, *P<0.05, **P<0.001, ***P<0.0001.

Supplementary Figure 4

Prostate cancer cells treated with alternative schedule of GLV-1h153 prior to external beam irradiation. Reduction of proliferative capability was measured by MTT assay at 24, 48 and 72 hours. Standard errors of the mean are shown. Significance is the result of 2-Way ANOVA with Bonferroni multiple comparisons test, *P<0.05, **P<0.001, ***P<0.0001.

Supplementary Figure 5

Viral gene expression in cells infected with GLV-1h153 4 hours after external beam irradiation. Viral β-galactosidase measured by CPRG assay at 48 and 72 hours post-treatment. Standard errors of the mean are shown. Significance is the result of 2-Way ANOVA with Bonferroni multiple comparisons test, *P<0.05, **P<0.001, ***P<0.0001.

Supplementary Figure 6

Viral gene expression in cells treated with alternative schedule of GLV-1h153 4 hours prior to external beam irradiation. Viral β-galactosidase measured by CPRG assay at 48 and 72 hours post-treatment. Standard errors of the mean are shown.

Supplementary Figure 7

Densitometry quantification of the western blot images presented in Figure 5C. Presented as percentage change compared to the baseline level seen in control samples.

Supplementary Figure 8

Biodistribution of ^131^I 7 days post-intratumoural injection of 1x10^6^ GLV-1h153 and 5 days post-treatment with 1mCi ^131^I.

Supplementary Figure 9

Biodistribution of viral gene expression. Detected by viral encoded Renilla luciferase bioluminescence following intratumoural administration of GLV1h153 in PC3 xenografts.

Supplementary Figure 10

Confocal images of H2Ax foci resulting from DNA double strand breaks in TRAMP-C3 cells treated with GLV-1h153 and 131I. Blue: DAPI, Green: Viral GFP, White: γH2Ax foci.

Supplementary Figure 11

Scarring at the location of healed pox lesions (Black arrows) on the tails of mice treated intravenously with GLV-1h153.

Supplementary Figure 12

Hematoxylin and eosin staining in tissues collected from the control group in the TRAMP mouse therapy experiment shown in Figure 7C.

Supplementary Figure 13

Hematoxylin and eosin staining in tissues collected from the VV-NIS treated group in the TRAMP mouse therapy experiment shown in Figure 7C.

Supplementary Figure 14

Hematoxylin and eosin staining in tissues collected from the VV-NIS + ^131^I treated group in the TRAMP mouse therapy experiment shown in Figure 7C.

Supplementary Figure 15

Immunohistochemical staining for Ki67 in tissues collected from the control group in the TRAMP mouse therapy experiment shown in Figure 7C.

Supplementary Figure 16

Immunohistochemical staining for Ki67 in tissues collected from the VV-NIS treated group in the TRAMP mouse therapy experiment shown in Figure 7C.

Supplementary Figure 17

Immunohistochemical staining for Ki67 in tissues collected from the VV-NIS + ^131^I treated group in the TRAMP mouse therapy experiment shown in Figure 7C.

Supplementary Figure 18

Immunohistochemical staining for probasin in tissues collected from the control group in the TRAMP mouse therapy experiment shown in Figure 7C.

Supplementary Figure 19

Immunohistochemical staining for probasin in tissues collected from the VV-NIS treated group in the TRAMP mouse therapy experiment shown in Figure 7C.

Supplementary Figure 20

Immunohistochemical staining for probasin in tissues collected from the VV-NIS + ^131^I treated group in the TRAMP mouse therapy experiment shown in Figure 7C.
